# Disrupting the tumor-associated TNKS–USP25 protein–protein interface in cancer: structural basis, druggability, and therapeutic opportunities

**DOI:** 10.1039/d6ra01334a

**Published:** 2026-07-06

**Authors:** Emadeldin M. Kamel, Sally Mostafa Khadrawy, Mohamed A. M. Ali, Noha A. Ahmed, Saleh Alkhedhairi, Faris F. Aba Alkhayl, Al Mokhtar Lamsabhi

**Affiliations:** a Chemistry Department, Faculty of Science, Beni-Suef University Beni-Suef 62514 Egypt drnohascience@science.bsu.edu.eg; b Department of Biology, College of Science, Imam Mohammad Ibn Saud Islamic University (IMSIU) Riyadh 11623 Saudi Arabia; c Physiology Division, Zoology Department, Faculty of Science, Beni-Suef University P.O. Box 62521 Beni-Suef Egypt; d Department of Medical Biosciences, College of Veterinary Medicine, Qassim University P.O. Box 6622 Buraydah 51452 Saudi Arabia; e Department of Medical Laboratories, College of Applied Medical Sciences, Qassim University Buraydah 51452 Saudi Arabia; f Departamento de Química and Institute for Advanced Research in Chemical Science (IAdChem), Facultad de Ciencias, Módulo 13, Universidad Autónoma de Madrid 28049 Madrid Spain

## Abstract

Tankyrases (TNKS1/2) are multi-domain poly(ADP-ribose) polymerases that regulate Wnt/β-catenin signaling and broader cellular programs through both catalytic activity and extensive protein–protein interaction (PPI) networks. While most tankyrase-directed drug discovery has focused on inhibiting the PARP catalytic site, an emerging alternative is to target tankyrase stability by disrupting its interaction with the deubiquitinase USP25. USP25 functions as a positive regulator of tankyrase abundance by counteracting ubiquitin-dependent turnover; consequently, blocking the TNKS–USP25 PPI can reduce tankyrase levels, stabilize pathway antagonists such as AXIN, and dampen Wnt transcriptional output. In this review, we discuss current understanding of tankyrase domain architecture with emphasis on ankyrin repeat clusters (ARC1/2/4/5) that recognize short tankyrase-binding motifs (TBMs), and we highlight why ARC5 is a particularly actionable node for intervention in the TNKS–USP25 axis. We summarize the structural basis of USP25 recruitment *via* a C-terminal TBM-like element, discuss ARC hotspot features that support ligandability, and provide a practical chemical-biology framework for validating PPI disruption using orthogonal assays (co-immunoprecipitation/proximity ligation, biophysics such as SPR/ITC, cellular target engagement, and displacement formats including FP/FRET). We then evaluate reported small-molecule disruptors, including C44 and UAT-B, as proof-of-concept agents that link ARC5-centered binding to tankyrase destabilization and antitumor phenotypes in prostate cancer and multidrug-resistant colorectal cancer models. Finally, we outline key challenges—selectivity across ARCs, off-target risk, and context-dependent biology—and propose future directions, including structure-guided optimization, improved cell-active chemotypes, and dual-mechanism strategies that combine PPI disruption with catalytic inhibition or targeted degradation approaches.

## Abstract 2

Disrupting the TNKS–USP25 protein–protein interface destabilizes tankyrase, restores AXIN mediated β-catenin destruction, and reveals ARC5-centered opportunities for drugging Wnt driven and multidrug-resistant cancers.

## Graphical abstract

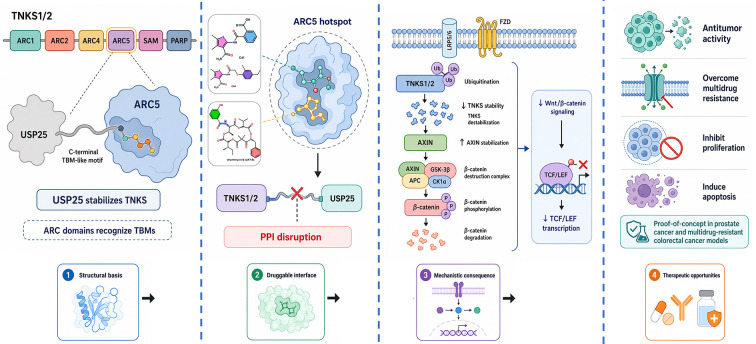

## 1. Introduction

**Fig. 1:**
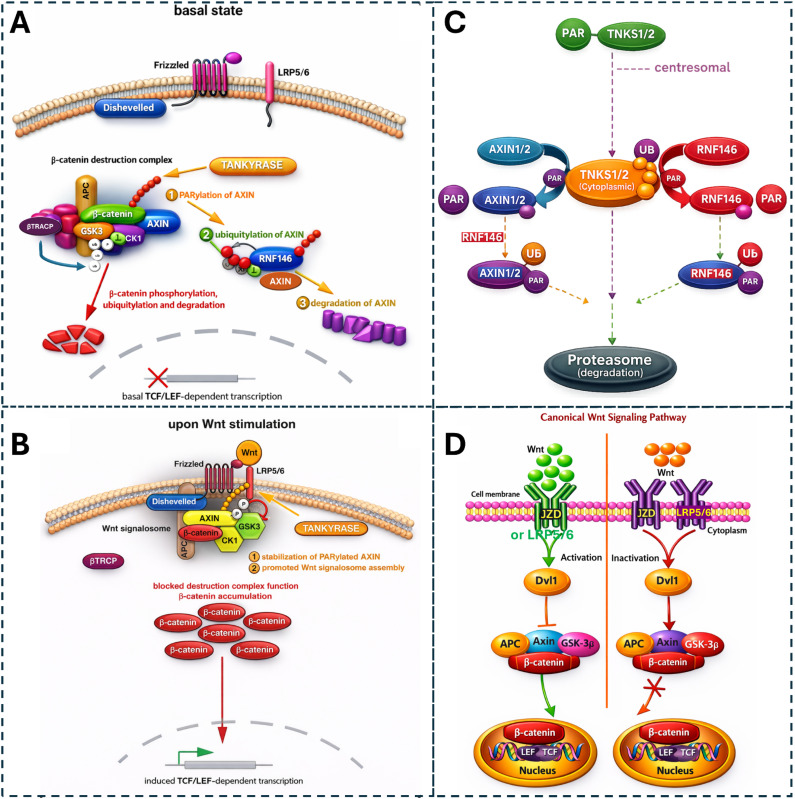
Tankyrase-dependent PARylation and RNF146-mediated ubiquitination regulate AXIN abundance and canonical Wnt/β-catenin signaling. (A) Basal (Wnt-off) conditions: the AXIN-centered destruction complex (with APC, GSK3β, and CK1) phosphorylates β-catenin, enabling βTrCP-dependent ubiquitination and proteasomal clearance, thereby maintaining low basal TCF/LEF transcription. In parallel, TNKS1/2 catalyze poly(ADP-ribosyl)ation of AXIN, which is recognized by the PAR-binding E3 ligase RNF146, promoting AXIN ubiquitination and proteasomal turnover, limiting cellular AXIN levels, reproduced from ref. 1 with permission from Wiley, copyright 2017. (B) Wnt-stimulated (Wnt-on) conditions: Wnt engagement of Frizzled and LRP5/6 activates Dishevelled and drives assembly of membrane-associated signalosomes that recruit AXIN and associated components. In this context, PARylated AXIN is stabilized and/or concentrated at the receptor complex, reducing destruction complex activity, allowing β-catenin accumulation and subsequent activation of TCF/LEF-dependent gene expression.^1^ (C) Proposed RNF146 feedback model: RNF146 couples PAR recognition to ubiquitination, targeting multiple PARylated proteins for degradation, including AXIN1/2 and TNKS1/2, and can also promote turnover of RNF146 itself—creating interconnected negative-feedback loops that tune pathway output across subcellular pools.^2^ (D) Pathway-level summary: canonical Wnt signaling is “on” when receptor activation suppresses β-catenin destruction, enabling nuclear β-catenin/TCF–LEF transcriptional programs; signaling is “off” when the destruction complex remains active (for example, when AXIN is elevated), preventing β-catenin-dependent transcription, reproduced from ref. 3, with permission from Elsevier, copyright 2020.

Aberrant activation of the Wnt/β-catenin pathway is a frequent driver of tumorigenesis and therapy resistance, especially in tumors carrying defects that compromise the β-catenin destruction machinery. A central brake on this pathway is AXIN, a scaffolding protein that nucleates the destruction complex and is widely viewed as a concentration-limiting factor for efficient β-catenin turnover. Tankyrase-1 and tankyrase-2 (TNKS1/2; PARP-5a/5b) enhance Wnt signaling in part by poly(ADP-ribosyl)ating (PARylating) AXIN, generating a PAR-dependent signal that recruits the PAR-binding E3 ligase RNF146 to promote AXIN ubiquitination and proteasomal degradation.^1–3^ Loss of AXIN destabilizes the destruction complex, allowing β-catenin to accumulate and drive TCF/LEF-dependent transcription, whereas Wnt stimulation can stabilize PARylated AXIN and promote its engagement at receptor-associated signalosomes—key steps summarized schematically in Fig. 1A–D.^4–6^

TNKS1/2 are multidomain enzymes that combine catalytic poly(ADP-ribose) polymerase activity with extensive protein-binding capacity. Their N-terminal ankyrin repeat region is organized into five ankyrin repeat clusters (ARCs); four of these (ARCs 1, 2, 4 and 5) contain conserved peptide-binding grooves that recruit diverse partners and substrates through short linear “tankyrase-binding motifs” (TBMs). Structural and sequence analyses have defined a canonical TBM core (often summarized as RXXΦDG, where Φ is a small hydrophobic residue) and revealed how subtle TBM variations, multivalency, and ARC selection combine to support substrate recognition. Fragment-based screening and biophysical work have further highlighted the ARC grooves as ligandable sites, supporting the idea that tankyrase scaffolding functions can be chemically modulated independently of catalytic inhibition.^7–9^

In cells, tankyrase abundance and signaling output are shaped not only by catalytic activity, but also by protein turnover controlled by the ubiquitin–proteasome system. The deubiquitinase USP25 has emerged as a direct positive regulator of tankyrases: USP25 binds TNKS proteins and counteracts their ubiquitination, increasing TNKS stability and thereby shifting the AXIN-β-catenin axis toward Wnt pathway activation. Importantly, structural characterization of the TNKS1 substrate-recognition region in complex with a USP25-derived binding segment has provided a concrete molecular template for how the TNKS–USP25 interaction is organized and how it might be disrupted pharmacologically.^10^

Most first-generation tankyrase chemical probes and drug-discovery programs have focused on inhibiting the TNKS catalytic domain; for example, the small molecule XAV939 suppresses β-catenin-mediated transcription by inhibiting TNKS1/2 and stabilizing AXIN. While catalytic inhibition remains a validated strategy, there is increasing interest in approaches that target catalysis-independent functions—such as ARC-mediated recruitment and signalosome assembly—with the aim of achieving distinct biological effects or improved therapeutic windows.^4,6,9^

Disrupting the TNKS–USP25 protein–protein interaction (PPI) represents a mechanistically attractive alternative that leverages the cell's own degradation machinery to reduce TNKS protein levels. Two small-molecule exemplars illustrate this concept: C44 was reported to selectively inhibit the TNKS–USP25 interaction, reduce TNKS stability, and attenuate proliferation of Wnt-pathway-dependent prostate cancer models; and UAT-B was shown to disrupt TNKS–USP25 complex formation, decrease TNKS levels, modulate Wnt/β-catenin signaling, and suppress growth of TNKS-overexpressing colorectal cancer models, including *in vivo* settings.^11,12^

Together, these advances motivate a focused examination of the TNKS–USP25 interface as a druggable regulatory node. In this review, we summarize the structural biology underlying TNKS substrate recognition and USP25 engagement, highlight experimental strategies used to detect and validate TNKS–USP25 disruption, and synthesize progress on chemical matter that targets this PPI. We also discuss open challenges—such as ARC selectivity, resistance mechanisms, and pathway-dependent liabilities—that will shape the development of next-generation TNKS scaffolding modulators.

## 2. Tankyrase domain architecture and substrate recruitment *via* ARCs

**Fig. 2:**
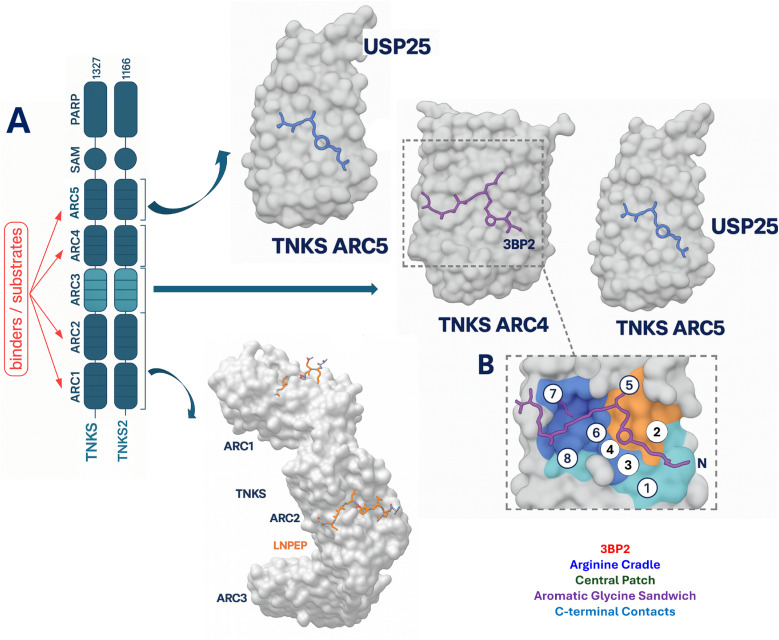
Tankyrase domain architecture and TBM-dependent substrate recruitment by ankyrin repeat clusters (ARCs), reproduced from ref. 15, with permission from Springer, copyright 2019. (A) Schematic domain organization of tankyrase-1 (TNKS) and tankyrase-2 (TNKS2), highlighting the N-terminal ankyrin repeat domain (25 ankyrin repeats arranged into five ankyrin repeat clusters, ARC1–ARC5) that mediates partner recruitment, followed by the SAM domain and the C-terminal PARP catalytic domain. Representative surface views illustrate TBM peptide engagement by individual ARCs, including ARC5 bound to a USP25-derived TBM and ARC4 bound to a 3BP2-derived TBM; an additional ARC1–ARC3 region is shown with a TBM peptide (LNPEP) to emphasize that multiple ARCs can function as peptide-binding modules within the extended ankyrin platform. (B) Enlarged view of the TBM-binding groove, showing the conserved interaction “hotspots” that coordinate TBM recognition (arginine cradle, central patch, aromatic glycine sandwich, and C-terminal contacts). Numbers indicate TBM residue positions (1–8), and the peptide N-terminus is marked.

Tankyrase-1 (TNKS; PARP5a) and tankyrase-2 (TNKS2; PARP5b) are multidomain PARP-family enzymes that integrate catalytic activity with protein-scaffolding functions. Both proteins contain a C-terminal ADP-ribosyltransferase/PARP catalytic domain, a sterile alpha motif (SAM) domain that supports tankyrase self-association, and an extended N-terminal ankyrin repeat region that mediates recruitment of binding partners. This N-terminal region consists of 25 ankyrin repeats organized into five ankyrin repeat clusters, designated ARC1–ARC5. Among these, ARC1, ARC2, ARC4, and ARC5 function as peptide-binding modules, whereas ARC3 lacks several key residues required for canonical tankyrase-binding motif recognition (Fig. 2A and B).^13–15^

### 2.1 ARC-dependent recognition of tankyrase-binding motifs

Tankyrase substrates and regulatory partners are commonly recruited through short linear tankyrase-binding motifs. Early mapping studies described these motifs as RxxPDG-like sequences, but subsequent peptide-library and structural analyses refined this view into a broader eight-residue recognition footprint. A practical consensus can be represented as:

### 2.2 R–x–x–[small hydrophobic residue or Gly]–[Asp/Glu]–Gly–[not Pro]–[Asp/Glu]

**Fig. 3:**
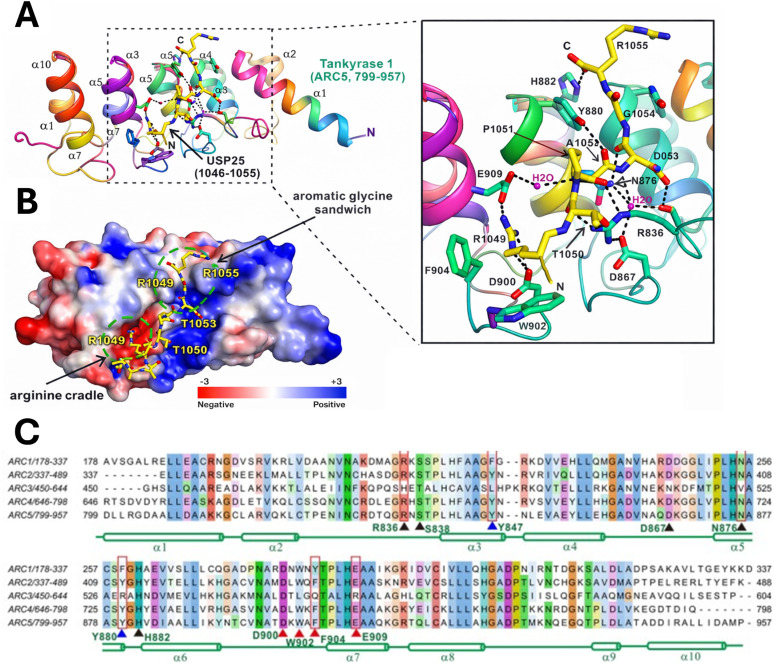
Structural determinants of TBM recognition by tankyrase ARCs and the basis for ARC-specific binding capacity.^10^. (A) Cartoon representation of the tankyrase-1 ankyrin repeat cluster 5 (ARC5) in complex with a USP25 tankyrase-binding motif (TBM) peptide, with a zoomed view highlighting the main intermolecular contacts that stabilize the peptide within the ARC groove. (B) Electrostatic surface depiction of ARC5 showing the TBM-binding pocket and the conserved hotspot features that anchor TBM peptides, including the basic “arginine cradle” accommodating the N-terminal Arg and the hydrophobic/aromatic pocket that enforces the Gly requirement (“aromatic glycine sandwich”). (C) Sequence comparison across ARC1–ARC5 illustrating conservation of residues that form the TBM-binding site in ARC1/2/4/5 and divergence at key positions in ARC3, consistent with reduced or absent canonical TBM binding by ARC3, reproduced from ref. 10, with permission from Cold Spring Harbor Laboratory Press, copyright 2017.

Within this motif, the N-terminal Arg and the conserved Gly are the most stringent determinants of binding. The Arg anchors the peptide within a conserved acidic pocket in the ARC groove, whereas the Gly is accommodated by a spatially restricted aromatic pocket. Other positions contribute to affinity and selectivity but tolerate greater sequence variation, explaining why tankyrases can recognize a broad set of substrates while still preserving a common binding logic (Fig. 3A and B).^7,13,14^ TBMs are most likely to function when located in flexible or intrinsically disordered regions, where they are accessible to the ARC grooves. This is consistent with the role of tankyrases as scaffold enzymes that engage multiple partners through relatively short, modular peptide elements rather than large folded interaction domains.

### 2.3 Functional differences among ARC1, ARC2, ARC4, ARC5, and ARC3

Although ARC1, ARC2, ARC4, and ARC5 share a conserved mode of TBM recognition, they are not functionally identical. Biochemical studies using isolated peptides and single-functional-ARC constructs show that individual ARCs can differ substantially in binding strength and substrate preference. ARC2 and ARC4 often show stronger binding to model TBM peptides, whereas ARC1 and ARC5 can display weaker or more context–dependent interactions.^7,13,14^ ARC3 is distinct from the other clusters because substitutions at key pocket residues disrupt the canonical recognition chemistry. As a result, ARC3 is generally considered a non-binding or “pseudo receptor” cluster. Its main contribution is likely architectural, helping maintain spacing and orientation within the extended ankyrin repeat platform rather than directly engaging canonical TBMs (Fig. 3C).

### 2.4 Structural principles of TBM recognition

Crystal structures of peptide-bound tankyrase ARCs show that TBM recognition is driven by a small number of conserved interface hotspots rather than by extensive side-chain complementarity across the whole peptide. These hotspots include the acidic pocket that binds the N-terminal Arg, the central region that accommodates the position-4 residue, the aromatic pocket that enforces the Gly requirement, and additional polar contacts near the C-terminal end of the motif. Together, these features allow tankyrase ARCs to recognize related but non-identical TBMs across different binding partners (Fig. 3A and B).^7,13,14^

This hotspot-based recognition mode has two important consequences. First, it explains why many different proteins can bind tankyrases using related short motifs. Second, it creates opportunities for selective regulation because subtle differences among ARCs, motif sequences, and surrounding residues can influence which ARC–TBM combinations are favored in a particular cellular context.

### 2.5 Multivalency and avidity in tankyrase recruitment

Tankyrase recruitment is frequently multivalent. A single tankyrase molecule contains several functional ARC binding sites, and SAM-domain-mediated self-association can increase the local concentration of these sites within higher-order assemblies. On the binding-partner side, many substrates either contain multiple TBMs or oligomerize, thereby presenting multiple tankyrase-binding elements simultaneously. These arrangements can produce avidity effects that stabilize complexes beyond what would be predicted from the affinity of a single isolated TBM–ARC interaction.^13,16^ AXIN provides an important example of this principle. Its tankyrase-binding region contains more than one TBM-containing segment, allowing bivalent or multivalent engagement of tankyrase ARCs. Such interactions help explain how tankyrase substrates can remain efficiently recruited despite the modest micromolar affinity of individual ARC–TBM contacts.^16,17^ Multivalency is therefore central to tankyrase biology. It influences substrate selectivity, sensitivity to mutations, and the likely behavior of inhibitors that target individual ARC pockets. Disrupting one binding site may weaken a complex without fully abolishing it, whereas compounds or mechanisms that interfere with multiple contacts, extended binding surfaces, or higher-order tankyrase assembly may have stronger functional effects.^10,13^

### 2.6 Relevance for targeting tankyrase protein–protein interactions

The ARC–TBM system provides the structural foundation for tankyrase substrate recruitment and for pharmacological strategies aimed at disrupting tankyrase protein–protein interactions. Because functional ARCs contain conserved but non-identical peptide-binding grooves, they present opportunities for both broad inhibition of tankyrase scaffolding and more selective targeting of individual ARC-dependent interactions. These principles are directly relevant to the TNKS–USP25 interface, whose molecular basis and small-molecule disruption are discussed in Section 4.

## 3. USP25 biology relevant to tankyrase regulation

### 3.1 USP25: a deubiquitinase whose activity is regulated in cells

Ubiquitin-specific protease 25 (USP25) is a cysteine protease deubiquitinase of the USP family. Like other deubiquitinases, USP25 can regulate protein abundance and signaling output by removing ubiquitin chains from specific client proteins, thereby opposing ubiquitin-dependent proteasomal degradation. Structural and biochemical studies indicate that USP25 activity is itself subject to regulation through oligomerization. The USP25 catalytic domain can form an autoinhibited homotetramer, and comparative analyses of USP25 and its paralog USP28 support a model in which USP25 exists in an auto-inhibited tetrameric state, whereas USP28 forms a constitutively active dimer.^18,19^ These findings are relevant to tankyrase regulation because they suggest that the effective activity of USP25 toward TNKS1/2 may depend not only on USP25 expression, but also on regulatory inputs that alter its oligomeric state or catalytic accessibility.^18,19^

### 3.2 USP25 stabilizes tankyrase by opposing ubiquitin-dependent turnover

Tankyrase abundance is controlled by the balance between ubiquitination, proteasomal degradation, and deubiquitination. Tankyrase-dependent PARylation can promote ubiquitin-mediated turnover through PAR-binding E3 ligases such as RNF146/Iduna, which recognizes PARylated substrates through its WWE domain and promotes their ubiquitination and degradation.^2,5,20^ This PARylation–ubiquitin crosstalk contributes not only to AXIN turnover but also to reciprocal regulation of tankyrase and RNF146 protein abundance.

Within this regulatory framework, USP25 was identified as a direct tankyrase-interacting deubiquitinase that stabilizes TNKS1/2. In cell-based studies, USP25 expression increased tankyrase protein levels and extended tankyrase half-life, whereas USP25 depletion or knockout reduced endogenous tankyrase abundance. This stabilizing effect required USP25 catalytic activity, as a catalytically inactive USP25 mutant failed to stabilize tankyrase. Consistently, USP25 expression reduced ubiquitinated tankyrase species in ubiquitination assays.^10^ Mechanistically, USP25 binds tankyrase through a short C-terminal tankyrase-binding motif-like element that engages the tankyrase ankyrin repeat region. Disruption of this interaction impairs USP25-dependent deubiquitination and stabilization of tankyrase, including protection from RNF146-driven tankyrase destabilization.^10^

### 3.3 USP25 promotes Wnt/β-catenin signaling through tankyrase stabilization

Because tankyrases promote AXIN turnover, USP25-dependent stabilization of tankyrase is expected to reduce AXIN abundance and enhance Wnt/β-catenin pathway output. Consistent with this model, USP25 knockout increased AXIN1 levels in HEK293T cells and reduced Wnt3a-induced SuperTopFlash reporter activity.^10^ USP25 knockdown or knockout also reduced Wnt3a-induced β-catenin accumulation and attenuated induction of the Wnt target gene AXIN2.^10^ Genetic and rescue experiments further support a tankyrase–AXIN-dependent mechanism. Depletion of AXIN partially rescued the Wnt reporter defect in USP25-null cells, indicating that increased AXIN contributes to reduced pathway output after USP25 loss. Re-expression of TNKS1 restored Wnt reporter activity in USP25-deficient cells, whereas a SAM-domain-defective TNKS1 mutant did not, suggesting that normal tankyrase scaffold behavior is required downstream of USP25.^6,10^ Reconstitution of USP25-null cells with wild-type USP25 rescued Wnt reporter activity, whereas tankyrase-binding-defective USP25 mutants failed to do so, supporting the conclusion that the USP25–tankyrase interaction is required for the Wnt-promoting effect of USP25.^10^

**Fig. 4:**
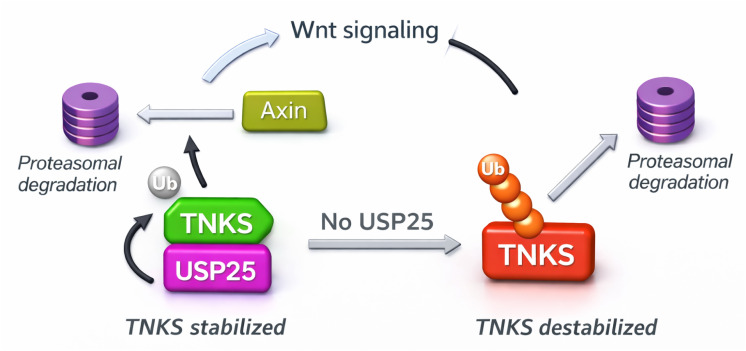
Working model for USP25-dependent stabilization of tankyrase and its impact on Wnt/β-catenin signaling.^10^ In cells expressing USP25, USP25 associates with tankyrase-1/TNKS2 and counteracts ubiquitin-dependent turnover of tankyrase, thereby maintaining tankyrase abundance. Elevated tankyrase activity promotes ubiquitin–proteasome–mediated degradation of Axin, relieving destruction-complex pressure and supporting Wnt pathway output. In the absence of USP25, tankyrase becomes polyubiquitinated and is degraded by the proteasome, leading to increased Axin stability and attenuation of Wnt signaling.

These pathway-level effects also translate into disease-relevant phenotypes. In the APC-mutant colorectal cancer cell line DLD-1, USP25 knockout reduced proliferation and colony formation. These effects were rescued by reintroducing TNKS1 or by depleting AXIN1/2, indicating that USP25 can support growth in Wnt-dependent cellular contexts through a tankyrase–AXIN axis.^6,10^ Together, these findings support a model in which USP25 promotes Wnt/β-catenin signaling primarily by stabilizing tankyrase. In the presence of USP25, tankyrase abundance is maintained, AXIN turnover is promoted, and β-catenin-dependent transcription is supported. In the absence of USP25, tankyrase undergoes increased ubiquitin-dependent degradation, AXIN becomes stabilized, and Wnt pathway output is reduced (Fig. 4).

### 3.4 Additional biological contexts connecting USP25 and tankyrase

**Fig. 5:**
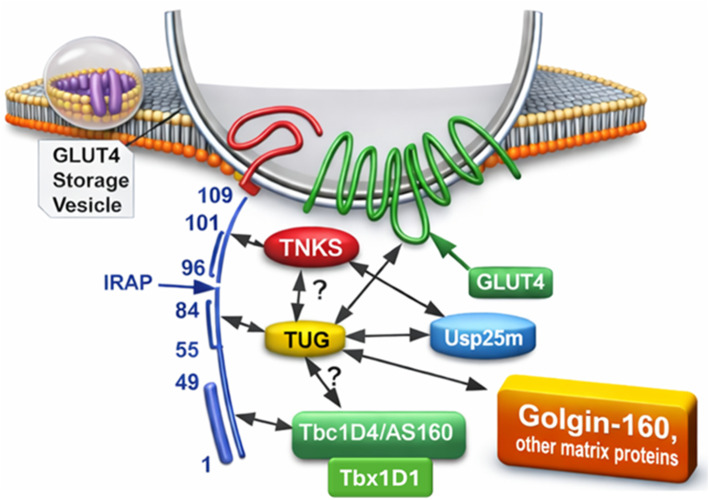
Proposed USP25–tankyrase module in insulin-responsive GLUT4 trafficking.^22^ Schematic of the GLUT4 storage vesicle (GSV) trafficking network in adipocytes highlighting a proposed interaction module linking tankyrase (TNKS) and the USP25 isoform Usp25m with GSV-associated regulators. TNKS is depicted within a protein network that includes the tethering factor TUG, the GSV cargo proteins GLUT4 and IRAP, and downstream signaling components Tbc1D4/AS160 and Tbx1D1, as well as Golgin-160 and other matrix proteins. Arrows indicate reported or proposed functional and/or physical connections among components that may coordinate vesicle retention and insulin-stimulated mobilization of GLUT4, reproduced from ref. 22, with permission from Frontiers, copyright 2022.

Although the USP25–tankyrase axis has been studied most extensively in the context of Wnt/β-catenin signaling, additional biological settings suggest that this regulatory relationship may have broader relevance. In insulin-responsive adipocytes, USP25 was reported to interact and colocalize with tankyrase in GLUT4-containing compartments. USP25 depletion reduced total GLUT4 levels and impaired insulin-stimulated glucose transport, linking the USP25–tankyrase interaction to membrane-trafficking phenotypes.^21^ A related trafficking context places tankyrase within a GLUT4 storage vesicle regulatory network that includes TUG, IRAP, and the AS160/Tbc1D4 axis. This model suggests that USP25–tankyrase coupling could influence GLUT4 compartment stability or insulin-responsive vesicle mobilization, although the precise mechanistic relationship between USP25 catalytic activity, tankyrase stability, and GLUT4 trafficking remains to be further defined (Fig. 5).^22^ Independent work in glioma cell models has also proposed that USP25 promotes invasion, migration, and proliferation by deubiquitinating TNKS1 and enhancing Wnt/β-catenin signaling.^23^ While additional validation across tumor types and experimental systems will be important, these findings are consistent with the broader concept that USP25-dependent tankyrase stabilization can act as a signaling-promoting node in selected disease contexts.

## 4. Molecular basis of the TNKS–USP25 interaction

### 4.1 USP25 C-terminal TBM-like element: minimal binding epitope and mutational validation

Mapping experiments revealed that USP25 engages tankyrase primarily through an extreme C-terminal tankyrase-binding motif-like sequence. The last seven residues of human USP25, R1049–R1055, contain the sequence RTPADGR, which conforms to the broader tankyrase-binding motif framework and is sufficient for direct binding to the tankyrase ankyrin-repeat region.^10^ Isothermal titration calorimetry showed that the full ankyrin-repeat region of TNKS1 binds the USP25 C-terminal heptapeptide with a dissociation constant of approximately 6.5 µM. A longer USP25 fragment spanning residues 720–1055 bound with a similar affinity of approximately 7.9 µM, indicating that the C-terminal residues provide the dominant binding epitope.^10^

Binding measurements with individual ARC-containing fragments showed that USP25 does not interact equally with all ARCs. ARC5 and ARC4 supported the strongest binding to the USP25 heptapeptide, with dissociation constants of approximately 7.1 µM and 9.3 µM, respectively. ARC2–3 and ARC1 bound more weakly, with reported dissociation constants of approximately 32.5 µM and 57.9 µM, respectively.^10^ These data suggest that USP25 can engage more than one ARC, while highlighting ARC5 and ARC4 as major contributors to the interaction.^7,10,14^ Cell-based mutagenesis confirmed the importance of the C-terminal motif. Substitution of key residues within the USP25 TBM-like sequence disrupted binding to TNKS1/2. In co-immunoprecipitation assays, R1049A, D1053A, and G1054A abolished the interaction, whereas T1050A reduced binding and S1048A had no detectable effect.^10^ These results establish the USP25 C-terminus as a compact but functionally essential binding element for tankyrase recognition.

### 4.2 Atomic basis of USP25 recognition by TNKS1 ARC5

**Fig. 6:**
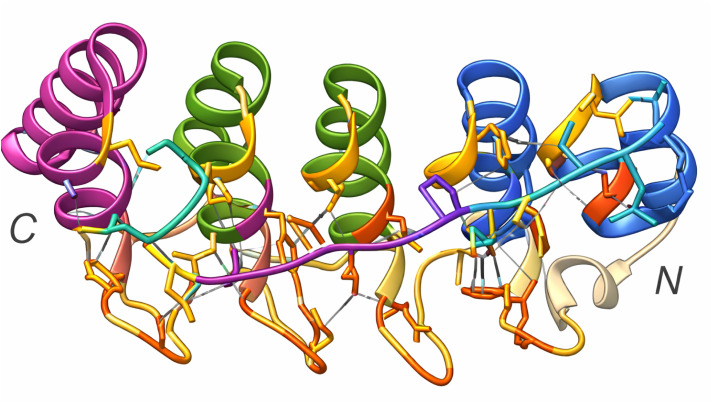
Conservation features mapped onto the TNKS1–USP25 binding interface. Ribbon view of TNKS1 ARC5 in complex with the USP25 C-terminal TBM peptide (PDB 5GP7). Residues classified as UMD positions are highlighted in red, and UME sites 11 and 12 are shown in orange; additional positions that tend to be shared among proteins that recognize related peptide substrates are indicated in dark cyan. The bound substrate motif is depicted in purple to emphasize that these conserved interface features recur in TBM-binding systems—for example across TNKS1/TNKS2, which engage RXXPDG-type motifs, and across other ankyrin-repeat binders such as RFXANK and ANRA2, which preferentially bind PXLPX[I/L] motifs.^24^

A high-resolution crystal structure of TNKS1 ARC5 bound to the USP25 C-terminal peptide provided an atomic view of the interaction. In this structure, the USP25 peptide adopts an extended conformation along the ARC5 binding groove, using the same core recognition features that support TBM binding by tankyrase ARCs more generally. However, the structure also reveals the USP25-specific contacts that explain the mutational and affinity data described above (Fig. 6).^10,24^ The N-terminal Arg of the USP25 motif, R1049, is anchored in the ARC5 arginine-recognition pocket. In ARC5, D900 and E909 form salt–bridge interactions with the guanidinium group of R1049, while W902 and F904 contribute hydrophobic and cation–π contacts that further stabilize the interaction.^10^ This explains why mutation of R1049 strongly disrupts tankyrase binding.

At the other end of the motif, G1054 is accommodated within the restricted glycine-recognition region of ARC5. The aromatic residues Y847 and Y880 pack near the peptide backbone, creating a spatially constrained environment that is incompatible with larger side chains. This structural constraint is consistent with the loss of binding observed for the G1054A mutant.^10^ Additional contacts stabilize the central and C-terminal portions of the peptide. N876 forms a hydrogen bond with the backbone carbonyl of D1053, and the terminal residue R1055 contributes C-terminal contacts, including interaction of the terminal carboxyl group with H882.^10^ Together, these contacts explain why the USP25 C-terminal motif behaves as a high-information binding element despite its short length. Consistent with the general ARC comparison discussed in Section 2, ARC3 lacks several conserved pocket residues required for canonical TBM recognition and is therefore unlikely to contribute directly to USP25 binding. For example, substitutions at positions corresponding to the ARC5 arginine-recognition and glycine-recognition features disrupt the geometry and electrostatics needed for productive peptide engagement.^10^

### 4.3 Ligandability of the ARC5 USP25-binding surface

The USP25-binding surface of ARC5 is not a flat or featureless protein–protein interface. Instead, it contains a set of chemically distinct subregions that coordinate the USP25 C-terminal motif. These include the arginine-recognition pocket, a central polar region, the glycine-recognition region, and C-terminal contact sites. Because these subregions create localized pockets and interaction hotspots, they provide a structural rationale for small-molecule targeting.^7,9,10^ Fragment-based screening studies support the ligandability of tankyrase ARC domains. Using differential scanning fluorimetry and NMR-based approaches, fragments were identified that bind ARC domains and compete with TBM peptides. Computational pocket and hotspot analyses localized favorable small-molecule binding regions to the TBM-binding groove, with prominent hotspots near the central patch and glycine-recognition region.^7,9^

Importantly, fragment binding depended on the integrity of residues that also contribute to peptide recognition. Mutation of residues in the glycine-recognition region abolished binding of a representative fragment, whereas mutation in the central patch reduced binding.^7,9^ These findings indicate that small molecules can engage functionally relevant sub-sites within the ARC groove rather than binding nonspecifically to the protein surface. For the TNKS–USP25 interaction, these observations are significant because they suggest that orthosteric PPI inhibitors can be designed to occupy parts of the USP25-recognition surface. Effective compounds may need to combine an anchor interaction in one hotspot with additional contacts across neighboring sub-sites to achieve sufficient potency, selectivity, and cellular activity.

### 4.4 Small-molecule disruption of the TNKS–USP25 interface

**Fig. 7:**
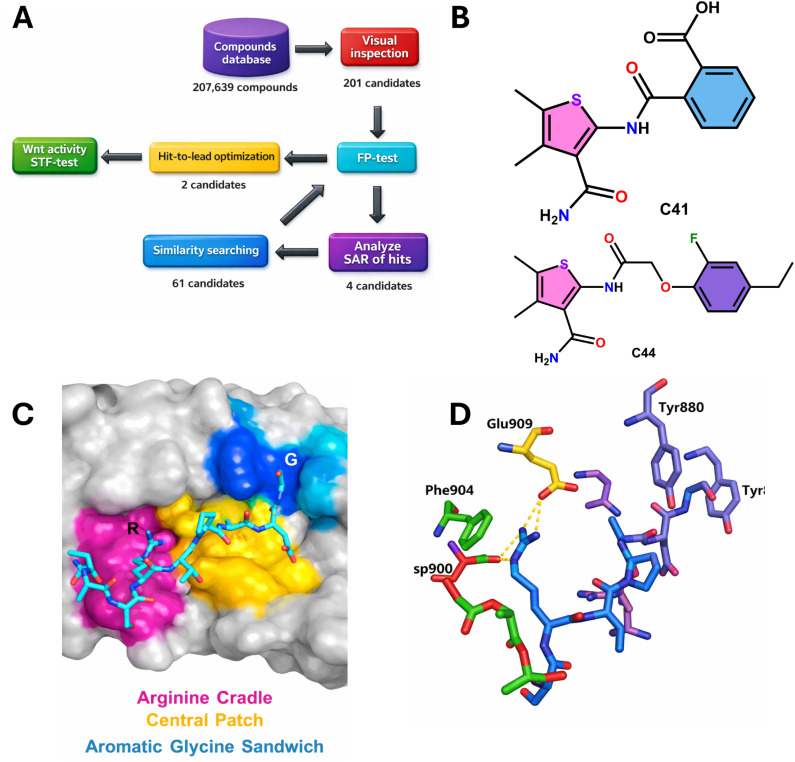
Structure-guided discovery of small molecules targeting the TNKS–USP25 interface at ARC5 by Cheng *et al.*^11^ (A) Overview of the hierarchical *in silico*-to-experimental screening workflow used to triage a >200 000-compound library to a small set of candidates for biophysical testing, structure–activity refinement, hit-to-lead optimization, and cell-based Wnt pathway readouts. (B) Chemical structures of the prioritized screening hits (including C41 and the lead compound C44). (C) Electrostatic surface representation of the TNKS ARC5 TBM-binding groove highlighting the conserved interaction hotspots—arginine cradle, central patch, and aromatic glycine sandwich—that accommodate key positions of the USP25 TBM (notably R1049 and G1054). (D) Close-up of the TNKS1 ARC5–USP25 peptide co-crystal structure (TNKS1 ARC5 residues 799–957 with USP25 residues 1046–1055; PDB 5GP7) illustrating the contact network at the interface; dashed lines denote representative hydrogen-bonding interactions within the arginine-cradle region reproduced from ref. 11, with permission from Elsevier, copyright 2019.

Two reported small-molecule approaches provide proof-of-concept that the TNKS–USP25 interaction can be disrupted pharmacologically through the ARC5 interface. The first approach used a hierarchical structure-guided virtual screening strategy to identify candidate binders of the TNKS–USP25 interface. Screening of more than 200 000 compounds led to prioritized hits, including C41 and the optimized lead compound C44. The design strategy was based on the known topology of the ARC5 TBM-binding groove and the USP25 peptide-bound structure, particularly the spatial arrangement of the key recognition features surrounding USP25 R1049 and G1054 (Fig. 7).^11^ Functionally, C44 was reported to disrupt the TNKS–USP25 interaction, reduce tankyrase stability, prolong AXIN half-life, and enhance β-catenin degradation. In prostate cancer models with Wnt-pathway dependence, C44 reduced cell proliferation and attenuated tumor growth *in vivo*.^11^ These findings support the idea that disrupting the USP25-dependent stabilization of tankyrase can indirectly suppress Wnt/β-catenin pathway output.

**Fig. 8:**
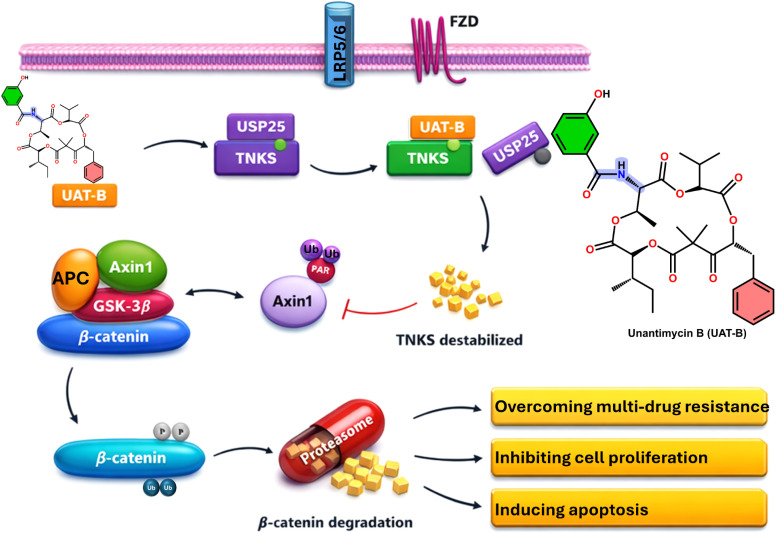
Proposed mechanism by which UAT-B disrupts the TNKS–USP25 axis to suppress Wnt/β-catenin signaling and overcome drug resistance.^12^ Schematic model illustrating that the neoantimycin analog UAT-B (unantimycin B) interferes with formation of the TNKS–USP25 complex, thereby promoting tankyrase destabilization and proteasomal degradation. Reduced tankyrase abundance stabilizes Axin1 and supports destruction-complex activity, leading to enhanced β-catenin phosphorylation/ubiquitination and proteasome-dependent β-catenin clearance, ultimately dampening Wnt pathway output. In TNKS-overexpressing colorectal cancer settings, this pathway inhibition is proposed to reduce proliferation, promote apoptosis, and improve multi-drug response.^12^

A second approach identified UAT-B, a neoantimycin analog, as an inhibitor of the TNKS–USP25 interaction in colorectal cancer models. Surface plasmon resonance experiments showed that UAT-B binds a TNKS1 fragment containing ARC5 with a dissociation constant of approximately 46.6 µM. Docking analysis suggested that UAT-B occupies part of the USP25-recognition surface and forms predicted interactions involving R836, N876, and K913.^12^ The involvement of N876 is notable because this residue also contributes to USP25 peptide recognition in the ARC5–USP25 structure. Mutation of N876 reduced UAT–B binding and abolished the ability of UAT-B to disrupt TNKS–USP25 complex formation, supporting an ARC5-centered binding mechanism 12. In cellular models, UAT-B decreased tankyrase levels, stabilized AXIN1, reduced β-catenin signaling, and suppressed growth of TNKS-overexpressing colorectal cancer cells, including in drug-resistant settings (Fig. 8).^12^

Together, C44 and UAT-B demonstrate that the TNKS–USP25 interface is pharmacologically tractable. However, both compounds require further optimization and independent validation before broader therapeutic conclusions can be drawn. Future inhibitors will likely need improved potency, selectivity among ARC pockets, favorable cellular exposure, and careful evaluation across Wnt-dependent and Wnt-independent disease contexts. These studies nevertheless establish ARC5-centered PPI disruption as a viable strategy for modulating tankyrase abundance through the USP25 regulatory axis.

## 5. Chemical biology and assays for TNKS–USP25 PPI

Because the TNKS–USP25 interface is mediated by binding of a short TBM-like segment in USP25 to the tankyrase ankyrin-repeat clusters (ARCs), chemical probes are expected to act as orthosteric competitors of the ARC peptide-binding groove. Robust interpretation therefore benefits from an assay cascade that separates: (i) direct binding/competition at isolated ARC domains, (ii) disruption of the endogenous interaction in cells, and (iii) downstream pathway and stability readouts.^10,12,14,25^

### 5.1 Assay strategy and key controls

A practical “confidence ladder” for disrupting the TNKS–USP25 protein–protein interaction begins with rapid, interference-aware fluorescence assays such as fluorescence polarization (FP) or Förster resonance energy transfer (FRET), then advances to orthogonal, label-free biophysical methods including surface plasmon resonance (SPR) and/or isothermal titration calorimetry (ITC), and finally moves into cellular validation using target-engagement approaches like CETSA alongside interaction readouts such as co-immunoprecipitation (co-IP) and proximity ligation assay (PLA).^14,25–30^ This staged strategy is applicable whether the starting point is fragments, virtual-screening hits, or optimized analogues. Across this cascade, several controls are recommended to ensure interpretability: binding-site controls should include tankyrase ARC mutant(s) that disrupt the peptide-binding groove (for example, the ARC5 N876A substitution that has been used as a pocket control in chemical-probe studies);^12^ ligand controls should incorporate unlabeled TBM peptide competitors or TBM-disrupting point mutants in USP25 (or within the peptide tracer) to verify sequence-specific binding;^10,14^ and assay-interference controls should be run in parallel as counter-screens to detect compound fluorescence or quenching, aggregation, detergent sensitivity, and nonspecific protein binding—an especially critical safeguard when using ARC-focused fluorescence screening formats.^25,26^

### 5.2 Co-immunoprecipitation (co-IP) and related pulldowns

Co-IP remains the most direct way to demonstrate that a candidate compound disrupts the TNKS–USP25 complex in cells. Typical designs use epitope-tagged TNKS and USP25 (or endogenous proteins, if antibodies are strong) with reciprocal IPs and IgG controls. For orthosteric competitors, compounds can be added either to live cells prior to lysis (preferred for target-engagement claims) or to lysates (useful for mechanism triage but more susceptible to dilution artifacts).^10–12^ Tankyrase-focused method collections recommend pairing co-IP of candidate substrates/binders with an orthogonal readout of tankyrase-dependent PARylation, so that loss of binding can be distinguished from broader perturbations of tankyrase activity or stability. For PPI-disruptors that destabilize TNKS by blocking deubiquitination, co-IP is often complemented by immunoblotting of TNKS abundance, ubiquitination state, and downstream substrates (*e.g.*, AXIN).^10,12,14^

Practical tips for co-IP when the goal is to quantify disruption:

Use TBM-pocket and TBM-sequence controls (ARC5 pocket mutant; USP25 TBM mutant; or excess TBM peptide) to anchor interpretation in binding-site specificity.^10,12^ Normalize for TNKS abundance: if compounds lower TNKS levels on the time scale of treatment, loss of co-IP signal may reflect reduced TNKS rather than reduced affinity. Shorter treatments (*e.g.*, 1–4 h) can help separate primary disruption from secondary turnover.^12^ Consider mild lysis conditions if endogenous complexes are fragile; however, maintain enough detergent/salt to suppress promiscuous ARC binding. Empirical optimization is typically required.^14^

### 5.3 Proximity ligation assay (PLA) for *in situ* PPI readout

PLA enables quantification of TNKS–USP25 proximity in intact cells and is valuable when co-IP is confounded by lysis-induced complex loss. In PLA, two primary antibodies raised in different species bind their targets, and proximity-dependent DNA probes generate a rolling-circle amplification product visualized as discrete fluorescent puncta.^31^ In the UAT-B chemical probe study, PLA was used alongside co-IP to show reduced TNKS1–USP25 proximity after compound treatment, and the ARC5 pocket-control mutant (N876A) was used to demonstrate binding-site dependence of the disruption phenotype.^12^

PLA controls that matter for TNKS–USP25: Single-antibody and no-primary controls to estimate background puncta; plus knockdown/knockout of one partner (or CRISPR tag) if feasible. TBM/pocket controls (USP25 TBM mutant; TNKS ARC5 pocket mutant) to ensure the PLA signal is reporting the specific interface of interest.^10,12^ Quantify puncta per cell across multiple fields and include cell-cycle or density covariates when relevant, as TNKS localization can vary with cellular state.^31^

### 5.4 SPR and ITC to quantify direct binding at ARC domains

SPR provides label-free, real-time binding kinetics and is a strong orthogonal follow-up to fluorescence assays. For ARC-peptide or ARC-small-molecule measurements, an ARC domain (or ARC module) is typically immobilized and analyte is injected as a concentration series; global fitting yields kinetic parameters and/or steady-state *K*_D_ values.^12,27^ In the UAT-B study, SPR was used to quantify binding of UAT-B to TNKS1 ARC5 and to show loss of detectable binding to the ARC5 N876A mutant, supporting an ARC5-pocket engagement model. Reported steady-state affinities were in the mid–high micromolar range (*e.g.*, ∼47 µM for UAT-B and ∼178 µM for an analogue).^12^ ITC complements SPR by directly measuring binding thermodynamics (Δ*H*, Δ*S*) and stoichiometry in solution. For weak binders or solubility-limited compounds typical of early PPI-disruptor discovery, careful buffer matching and (when needed) displacement formats can extend the measurable affinity range.^28^

Common pitfalls and mitigations for ARC biophysics: protein stability: ARC multi-domain constructs can require elevated salt (*e.g.*, ≥300 mM NaCl for ARC2–3 and ARC1–5) and sometimes glycerol; instability can masquerade as binding anomalies.^14^ Surface artifacts in SPR: confirm that regeneration conditions do not denature the ARC domain; compare immobilization strategies and include blank/reference channels.^27^ Small-molecule solubility: use matched DMSO, monitor for refractive-index artifacts (SPR) and heats of dilution (ITC), and avoid concentrations that trigger precipitation.^27,28^

### 5.5 CETSA for cellular target engagement

CETSA exploits the principle that ligand binding often increases the thermal stability of a target protein in cells or lysates. After compound treatment, samples are heated across a temperature gradient (or at an isothermal point for dose–response), soluble protein is quantified (immunoblot, MS), and a stabilization curve is derived as an engagement metric.^29,30^ For TNKS–USP25 PPI inhibitors, CETSA provides an especially useful orthogonal readout because it can demonstrate ARC-pocket engagement even when the downstream effect is reduced TNKS abundance. In the UAT-B study, a TNKS1 CETSA temperature-gradient experiment (≈38–48 °C) and an isothermal dose–response format (*e.g.*, at 42 °C) were used to support cellular engagement.^12^

Recommended CETSA workflow (immunoblot-based): Choose the format: (i) melt-curve CETSA to select a working temperature window, then (ii) isothermal dose–response (ITDR-CETSA) for potency ranking.^30^ Use matched vehicle controls, and include a negative control compound (or ARC-pocket mutant line if available) to distinguish general proteostasis effects from specific stabilization.^12,29^ Where possible, use orthogonal engagement (*e.g.*, SPR/FP) to interpret ambiguous CETSA shifts, as some ligands can destabilize targets or shift apparent solubility through indirect mechanisms.^29,30^

### 5.6 Fluorescence polarization (FP) and FRET displacement assays

FP is well suited for quantifying ARC–TBM binding and for screening orthosteric competitors. A widely used format titrates ARC protein against a fixed, low concentration of fluorescein-labeled TBM peptide (*e.g.*, 25 nM), measuring polarization as a function of complex formation. In tankyrase protocol collections, ARC proteins are commonly tested as single domains or ARC modules with buffer conditions that include HEPES, TCEP, and a mild detergent such as CHAPS to reduce nonspecific binding.^14,26,32^ The same setup can be converted into a displacement assay by pre-forming an ARC-tracer complex (ARC near its tracer KD) and titrating test compounds; loss of polarization reports competition. Because small-molecule libraries can contain fluorescent or quenching compounds, best practice is to include parallel intensity readouts and/or multi-wavelength counterscreens.^25,26,32^ FRET assays provide an alternative homogeneous format that can be more tolerant of certain tracer designs and can be engineered for high-throughput screening. A published tankyrase-focused platform uses FRET to screen for small molecules that disrupt ARC-mediated PPI scaffolding functions, with confirmatory steps to manage fluorescence interference and promiscuous binders.^25^

Key design choices for TNKS–USP25 FP/FRET assays: Tracer design: a minimal TBM octapeptide plus short flanks can maximize dynamic range; include a linker (*e.g.*, β-Ala) between fluorophore and peptide to reduce steric effects.^14,26,32^ Protein format: start with ARC5 for USP25; then test across ARCs (and TNKS1 *vs.* TNKS2) for selectivity profiling.^12,14^ Data analysis: use appropriate binding models (single-site or competition) and report both apparent IC_50_ and *K*_*i*_ (where tracer *K*_D_ is known).^26,32^

### 5.7 Recommended workflow for TNKS–USP25 PPI-disruptor discovery

An assay cascade that has proven informative in tankyrase scaffold-targeting work combines: (1) biochemical competition at ARC domains, (2) orthogonal binding confirmation, and (3) cellular engagement plus interaction disruption. Below is a practical, decision-oriented sequence tailored to TNKS–USP25.^11,12,25^

• Primary screen: FP or FRET competition using ARC5 and a fluorescent USP25 TBM peptide tracer. Include intensity counterscreens and detergent controls early.^14,25,26^

• Hit confirmation: SPR against ARC5 (plus ARC5 pocket mutant control) to verify direct binding and map to the TBM groove; triage for solubility/stability artifacts.^12,27^

• Selectivity: test binding/competition across ARC modules and TNKS1 *vs.* TNKS2; consider counterscreens *versus* unrelated ankyrin-repeat proteins to assess promiscuity.^14,27^

• Cellular engagement: CETSA (melt curve → ITDR) on TNKS1/2 after compound treatment to support intracellular target engagement.^12,29,30^

• Cellular PPI disruption: co-IP and/or PLA of TNKS–USP25 (ideally endogenous). Use pocket/TBM mutants as specificity controls.^10,12,31^

• Mechanistic biology: monitor TNKS ubiquitination/turnover and downstream pathway markers (AXIN levels, Wnt/β-catenin transcriptional output) to connect engagement to function.^10,12^

## 6. Small-molecule disruption of TNKS–USP25

### 6.1 Rationale: blocking USP25 recruitment as an alternative to catalytic tankyrase inhibition

Most tankyrase inhibitors developed to date target the C-terminal PARP catalytic domain and suppress PARylation. However, multiple studies indicate that catalytic inhibition can increase cellular tankyrase protein abundance, and that in some Wnt-driven settings (especially when TNKS protein levels are high) catalytic inhibition may be insufficient to durably suppress β-catenin-dependent transcription. By contrast, disrupting the TNKS–USP25 interaction aims to prevent USP25-mediated deubiquitination and stabilization of TNKS, thereby promoting TNKS turnover, stabilizing Axin, and shutting down Wnt pathway output through a distinct (degradation-centered) mechanism.^10,12,33^

From a chemical-biology perspective, this strategy is attractive because the USP25-binding surface resides in the ankyrin repeat cluster (ARC) region—absent from other PARP family members—offering a route to higher target selectivity than pan-PARP catalytic inhibitors.^33^

### 6.2 C44: first reported small-molecule disruptor of the TNKS–USP25 PPI

C44 was identified *via* hierarchical structure-based virtual screening of >200 000 compounds against the experimentally characterized USP25/TNKS-ARC5 complex. The authors reported that C44 binds the TNKS–USP25 protein–protein interface and disrupts complex formation *in vitro*, consistent with a competitive mechanism at (or near) the ARC5 peptide-binding surface.^11^ Mechanistically, disrupting USP25 recruitment decreased functional stabilization of tankyrase and led to an increased half-life of Axin and breakdown of β-catenin, linking the PPI disruption directly to canonical Wnt pathway suppression.^11^ Reported prostate cancer phenotypes included reduced proliferation *in vitro* and reduced tumor growth *in vivo* after selective inhibition of the TNKS–USP25 interaction by C44. Publicly accessible summaries additionally note activity in commonly used prostate cancer cell lines (PC-3 and LNCaP).^11,34^ Because the Cancer Letters article is not fully open access, many quantitative parameters (*e.g.*, binding affinity, cellular EC_50_/IC_50_, *in vivo* dosing, and the extent of on-target rescue by ARC5-pocket mutants) are not readily extractable from the public abstract alone. Nevertheless, C44 established proof-of-principle that the TNKS–USP25 interaction is chemically tractable and that disrupting it can produce anti-tumor phenotypes consistent with Wnt pathway suppression.^11,35^

### 6.3 UAT-B: a neoantimycin analog that engages ARC5 and overcomes MDR in CRC models

Unantimycin B (UAT-B) is an antimycin-type depsipeptide (neoantimycin analog) isolated from *Streptomyces* conglobatus. In colorectal cancer (CRC) systems, UAT-B was shown to disrupt the TNKS–USP25 complex, decrease TNKS protein levels, stabilize Axin 1, suppress Wnt/β-catenin signaling, and trigger apoptosis.^12^

Evidence chain for direct target engagement and PPI disruption:

• Cellular co-IP and proximity ligation assay (PLA) showed reduced TNKS–USP25 association after UAT-B treatment.^12^

• Surface plasmon resonance (SPR) demonstrated direct binding of UAT-B to a TNKS1 ARC5-containing fragment (residues 799–981) with *K*_D_ ≈ 46.62 µmol L^−1^.^12^

• A cellular thermal shift assay (CETSA) supported UAT-B engagement of TNKS in cells.^12^

• Site-directed mutagenesis mapped UAT-B sensitivity to the ARC5 pocket: N876A markedly reduced binding and abrogated the ability of UAT-B to block the USP25/TNKS interaction.^12^

• A methylated analog (UAT-Bp) designed to remove a key hydrogen-bonding feature showed substantially weaker binding (*K*_D_ ≈ 178 µmol L^−1^), reinforcing a specific pharmacophore–pocket interaction model.^12^

Functionally, UAT-B displayed a strong dependence on TNKS abundance and a TNKS-dependent Wnt program. Across a panel including 12 CRC cell lines and non-malignant colorectal cells, higher TNKS protein levels correlated with greater UAT-B potency (Pearson *r* = −0.7459, *P* = 0.0034, *n* = 13). Normal colorectal cell lines showed much higher IC_50_ values than a sensitive CRC line (SW480), suggesting a potential therapeutic window.^12^ Mechanistic validation experiments indicated that TNKS depletion blunted UAT-B-induced TOPFlash reporter inhibition, β-catenin degradation, reduced cell viability, and apoptosis, supporting an on-target dependence on tankyrase-mediated Wnt signaling in sensitive cells.^12^

*In vivo* efficacy emphasized the MDR setting and ARC5-driven target dependence:

• In SW480 cell line-derived xenografts (high TNKS), daily UAT-B suppressed tumor growth dose-dependently (*T*/*C* ≈ 46.24% at 0.5 mg kg; ≈ 22.96% at 1 mg kg^−1^). In an RKO xenograft model (lower TNKS), UAT-B showed minimal effect.^12^

• In an HCT-8/5-FU multidrug-resistant xenograft model, UAT-B alone (1 mg kg^−1^) strongly delayed tumor growth (*T*/*C* ≈ 18.22%) and combination with 5-FU (UAT-B 0.5 mg kg^−1^ + 5-FU 10 mg kg^−1^) retained strong activity without obvious weight loss; G007-LK (a catalytic TNKS inhibitor) was ineffective in this MDR model.^12^

• In a patient-derived xenograft (PDX) CRC model, UAT-B (1 mg kg^−1^, once daily for 35 days) inhibited tumor growth (*T*/*C* ≈ 38.45%) and reduced TNKS/β-catenin and Wnt target proteins (including *C*-myc and Cyclin D1) while increasing apoptosis markers.^12^

• UAT-B also suppressed tumorigenesis/progression in APCmin/+ spontaneous CRC models, consistent with Wnt-pathway antagonism *in vivo*.^12^

### 6.4 Comparative lessons and translational considerations

Together, C44 and UAT-B provide complementary proof-of-concept for small-molecule disruption of the TNKS–USP25 interaction (Table 1). C44 illustrates a purely structure-guided, synthetic screening route to a PPI inhibitor in prostate cancer contexts, whereas UAT-B demonstrates how an ARC5-pocket binder can be linked to a biomarker-driven (high-TNKS) response and robust *in vivo* activity in MDR CRC models.^11,12^

**Table 1:** Comparative overview of the TNKS–USP25 PPI disruptors C44 and UAT-B

Feature	C44	UAT-B
Discovery origin	Structure-based virtual screening (>200 k) on USP25/TNKS-ARC5 complex	Natural product (neoantimycin analog) isolated from *Streptomyces* conglobatus
Primary mechanism	Disrupts TNKS–USP25 association; destabilizes TNKS; increases Axin half-life and β-catenin breakdown	Disrupts TNKS–USP25 association; promotes TNKS degradation; stabilizes Axin 1; suppresses Wnt signaling; induces apoptosis
Direct binding evidence to ARC5	Reported binding at the PPI interface (details in abstract; quantitative affinity not in public summary)	SPR *K*_D_ ≈ 46.62 µM to TNKS1(799–981); CETSA supports engagement; N876A reduces binding
Disease models emphasized	Prostate cancer (reported *in vitro* and *in vivo*)	CRC, with emphasis on TNKS-high and MDR settings; includes PDO, CDX, PDX, APCmin/ + models
Key translational hook	First proof-of-principle PPI inhibitor for this interface	Biomarker dependence (TNKS-high) and strong MDR *in vivo* efficacy; provides pharmacophore and pocket-residue map

For future drug discovery, three practical takeaways emerge: (i) ARC5 pocket engagement can be tied to clear genetic/biophysical controls (*e.g.*, N876A pocket mutation and pharmacophore edits), enabling strong on-target validation; (ii) efficacy may be enriched in tumors with high TNKS protein and a TNKS-dependent Wnt program, suggesting patient stratification strategies; and (iii) medicinal chemistry will likely be needed to improve affinity, exposure, and developability, particularly given the mid-micromolar binding constants reported to date.^12^

### 6.5 Pyrrolone-based ARC4 inhibitors as additional evidence for ARC-domain druggability

In addition to C44 and UAT-B, recent work has expanded the chemical space for targeting tankyrase scaffolding functions through the discovery of pyrrolone-based inhibitors of the ARC peptide-binding domains. Bosetti *et al.* used a FRET-based screening platform against EU-OPENSCREEN libraries to identify pyrrolone compounds that inhibit ARC-mediated peptide recognition. The optimized compound S8, also termed ARCher-142, binds selectively to the TNKS2 ARC4 peptide-binding domain with a reported potency of approximately 8 µM. NMR and X-ray crystallographic analyses defined the binding site and provided a structural rationale for ARC4 selectivity, while cellular experiments showed that S8/ARCher-142 can attenuate WNT/β-catenin signaling.^36^ Although these pyrrolone-based inhibitors have not been reported to directly disrupt the TNKS–USP25 interaction, they are highly relevant to the present review because they demonstrate that individual tankyrase ARC domains can be selectively and structurally targeted by small molecules. This finding strengthens the broader concept that ARC-mediated scaffolding interactions are druggable and suggests that future medicinal chemistry may enable the development of ARC-selective or multi-ARC ligands capable of modulating specific tankyrase PPIs, including the TNKS–USP25 axis.^36^

## 7. How PPI disruption compares to catalytic inhibition

Tankyrase (TNKS/TNKS2) function can be perturbed either by blocking its PARP catalytic site (the dominant approach for Wnt-pathway suppression) or by disrupting specific protein–protein interactions (PPIs) that recruit or stabilize TNKS. While both strategies can converge on reduced Wnt/β-catenin transcription, they differ markedly in what they do to TNKS protein abundance, substrate handling, and non-catalytic scaffolding outputs. This section outlines mechanistic expectations and experimental discriminators for TNKS–USP25 PPI disruption *versus* catalytic inhibition.

### 7.1 What is being inhibited: enzyme activity *versus* a regulatory interaction

Catalytic TNKS inhibitors (TNKSi; *e.g.*, XAV939 or WIKI4) target the PARP domain and suppress poly(ADP-ribosyl)ation of TNKS substrates such as AXIN, thereby preventing PAR-dependent ubiquitination (PARdU) and proteasomal turnover of these substrates.^4,5,37^ Mechanistically, TNKS PARylation creates a binding signal for RNF146/Iduna (WWE-domain recognition of PAR), which then ubiquitinates PARylated targets; this paradigm was defined for AXIN and extended to other TNKS-dependent substrates.^5,20^ By contrast, TNKS–USP25 PPI disruptors (*e.g.*, C44 and UAT-B) are designed to block recruitment of USP25 to the ARC5 substrate-binding pocket without directly targeting the catalytic site. In cell-based systems, these compounds are reported to destabilize TNKS and dampen downstream Wnt outputs, consistent with removal of a deubiquitinase-based “protection” mechanism rather than inhibition of PARylation chemistry *per se*.^12,38^

### 7.2 Expected consequences for TNKS abundance and pathway wiring

A key practical distinction between tankyrase catalytic inhibition and TNKS–USP25 PPI disruption is the direction of change in cellular TNKS protein levels. Because TNKS can auto-PARylate and thereby trigger its own PARdU-dependent degradation, catalytic inhibition often stabilizes TNKS proteins and can increase their cellular abundance, even while suppressing enzymatic output [2, 4, 39]. By contrast, disrupting the TNKS–USP25 interaction is expected to decrease TNKS abundance by preventing USP25-mediated deubiquitination and allowing ubiquitin ligases and proteasomal turnover to proceed.^12,38^

These opposing effects on TNKS abundance have important downstream consequences for pathway wiring. Under catalytic tankyrase inhibition, substrates—most notably AXIN—are stabilized because PARylation and subsequent RNF146-dependent ubiquitination are blocked.^4,5^ This also alters RNF146 signaling: RNF146 recognizes PAR as a degron through its WWE domain and is allosterically activated by PAR/iso-ADPr, so catalytic inhibition reduces the PAR cue itself, whereas USP25 PPI disruption leaves the PAR signal intact but shifts the balance toward ubiquitin-mediated destruction of TNKS.^5,20^ In functional terms, both strategies can suppress Wnt/β-catenin transcriptional outputs in APC-mutant contexts, but the kinetics and depth of suppression may differ because the molecular rewiring is not the same—AXIN stabilization occurs alongside elevated but inactive TNKS with TNKSi, *versus* AXIN stabilization coupled to reduced TNKS abundance with PPI disruption.^4,12,38^ Finally, catalytic TNKS inhibition can elicit feedback and compensation, including changes in destruction-complex composition and, in some cell types, the formation of large AXIN/TNKS-containing puncta (“degradasomes”).^39^

### 7.3 Scaffolding and condensate-like behavior: why catalytic inhibition may not phenocopy TNKS loss

Tankyrase is not only an enzyme; it is also a modular scaffold with multiple ankyrin-repeat clusters (ARCs) for substrate binding and a SAM domain that can polymerize. Structural and cell-based work indicates that SAM-dependent polymerization supports TNKS localization to Wnt destruction complexes and can amplify substrate engagement *via* avidity; importantly, polymerization can influence both catalytic and non-catalytic functions.^6,40^ Because TNKSi can stabilize TNKS proteins while leaving ARC-mediated binding and SAM polymerization intact, catalytic blockade can preserve (or even accentuate) TNKS scaffolding behavior. In Wnt-pathway settings, TNKSi treatment has been associated with formation of large AXIN/TNKS-positive assemblies (“degradasomes”), which are thought to be dynamic destruction-complex structures.^6,39^

This creates a conceptual scenario in which enzymatically inactive but abundant TNKS could still nucleate or reorganize interaction networks. A recent reviewed preprint proposes that catalytic inhibition-driven TNKS accumulation in destruction complexes can alter complex dynamics and β-catenin turnover, and that strategies causing TNKS degradation may suppress Wnt signaling without the same puncta formation.^41^ From this viewpoint, TNKS–USP25 PPI disruption (which decreases TNKS abundance) should more closely resemble genetic depletion of TNKS1/2 than catalytic inhibition does, with greater likelihood of extinguishing both catalytic activity and scaffolding-dependent functions. However, the extent to which particular phenotypes depend on catalysis *versus* scaffolding is substrate- and context-dependent.^6^

### 7.4 Translational and safety considerations: on-target liabilities may differ in degree, not kind

In APC-mutant colorectal cancer models, TNKS catalytic inhibition shows clear pathway activity but has been limited by gastrointestinal (GI) toxicity in preclinical studies, consistent with the central role of Wnt signaling in intestinal homeostasis.^42,43^ Improved tolerability has been reported for some newer TNKS-selective inhibitors, but it remains an open question whether PPI-disruption strategies that reduce TNKS abundance will materially shift the therapeutic window *versus* catalytic inhibition, because both ultimately dampen Wnt/β-catenin output.^43^

**Fig. 9:**
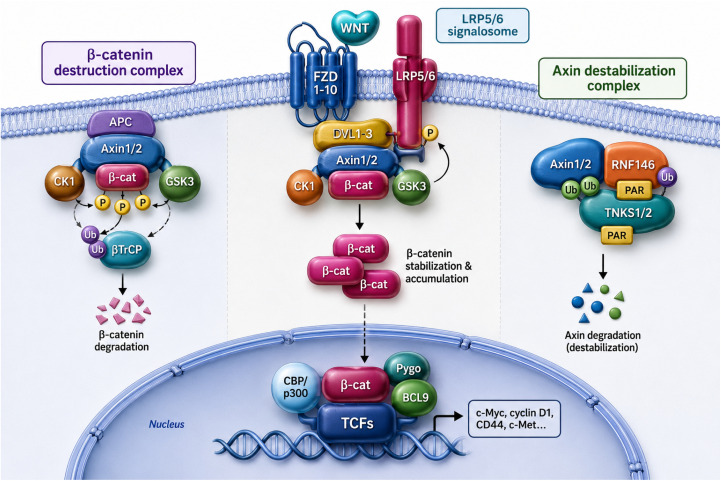
Tankyrase-dependent control of AXIN abundance in canonical Wnt/β-catenin signaling. In the Wnt “on” state, secreted Wnt ligands engage Frizzled (FZD) and LRP5/6, promoting assembly of an LRP5/6 signalosome and downstream signaling that supports β-catenin accumulation, nuclear entry, and activation of TCF/LEF-dependent transcription. Under basal conditions, β-catenin levels are restrained by the destruction complex (centered on AXIN1/2 with APC, CK1, and GSK3), which phosphorylates β-catenin to enable βTrCP-mediated ubiquitination and proteasomal degradation. AXIN is widely viewed as a concentration-limiting scaffold for destruction-complex activity, and TNKS1/2 promotes Wnt pathway output by PARylating AXIN, creating a signal that can recruit the PAR-binding E3 ligase RNF146 to drive AXIN ubiquitination and turnover. Consequently, tankyrase inhibition stabilizes AXIN, enhances β-catenin degradation, and reduces downstream transcriptional responses.^44^

As summarized in Fig. 9, canonical Wnt signaling is controlled by the balance between β-catenin destruction and ligand-driven signalosome assembly at Frizzled/LRP5/6. In the absence of Wnt, the AXIN-centered destruction complex (with APC, CK1, and GSK3) phosphorylates β-catenin to promote βTrCP-dependent ubiquitination and proteasomal degradation, thereby limiting TCF/LEF-mediated transcription. Tankyrases (TNKS1/2) enhance pathway output by promoting AXIN turnover—through PARylation of AXIN and recruitment of the PAR-directed E3 ligase RNF146—which weakens destruction-complex capacity and permits β-catenin accumulation. This mechanism provides a clear rationale for tankyrase inhibition strategies: stabilizing AXIN restores destruction-complex activity, reduces β-catenin levels, and attenuates Wnt target gene expression.^44^

A potentially favorable feature of ARC-targeting PPI inhibitors is selectivity: ARCs are unique to tankyrases, whereas the PARP catalytic fold is shared across PARP family members. Fragment-based ARC screening campaigns have explicitly highlighted the need for substrate-binding antagonists as chemical probes to disentangle catalytic and scaffolding functions.^9^

### 7.5 Practical experimental discriminators and recommended workflow

When evaluating TNKS–USP25 PPI disruptors, it is useful to run a side-by-side “mechanism panel” alongside a reference catalytic tankyrase inhibitor (TNKSi), such as XAV939 or WIKI4, to distinguish direct PARP catalytic blockade from compound-induced TNKS destabilization.^4,37^ A first discriminator is TNKS abundance and ubiquitination: TNKS1/2 levels can be monitored by immunoblotting after compound treatment across a time course, since catalytic inhibitors frequently increase TNKS abundance, whereas TNKS–USP25 disruptors are expected to reduce it. Where feasible, ubiquitin pull-downs or TUBE enrichment can further test the prediction that PPI disruption increases TNKS ubiquitination by preventing USP25-mediated deubiquitination and permitting proteasomal turnover.^12,38,45^

In parallel, catalytic engagement should be assessed directly using in-cell PARylation readouts, such as anti-PAR immunoblotting of immunoprecipitated TNKS or substrate PARylation proxies, to determine whether the compound inhibits tankyrase PARP activity. Importantly, a “pure” PPI disruptor could leave TNKS catalytic activity intact *in vitro* yet still reduce overall cellular PARylation outputs indirectly by lowering TNKS abundance, so separating these possibilities experimentally is essential.^4,6,20^

Downstream pathway consequences can then be profiled by measuring AXIN stabilization and destruction-complex remodeling. This includes quantifying AXIN1/2, β-catenin levels, and β-catenin/TCF reporter activity, while also monitoring AXIN/TNKS puncta by immunofluorescence as a marker of TNKSi-induced “degradasomes.” Because loss of TNKS protein is not expected to phenocopy puncta induction, this imaging readout can help differentiate catalytic inhibition-driven remodeling from PPI disruption-driven depletion.^4,39,41^

Finally, mechanism assignment should be strengthened by genetic tests of target dependence. Combining compound treatment with TNKS1/2 or USP25 knockdown/knockout enables epistasis analysis: if a compound acts primarily through disrupting the TNKS–USP25 interaction, loss of USP25 should diminish any incremental compound effect, while loss of TNKS should ablate it.^12,38^ Collectively, this workflow helps ensure that an apparent TNKS–USP25 PPI inhibitor is not an unrecognized catalytic TNKSi (or a nonspecific proteostasis stressor), and it provides an interpretable molecular “fingerprint” for how Wnt signaling suppression is achieved.

### 7.6 Clinical status of TNKS–USP25 disruptors and other tankyrase-directed agents

At present, no clinical trials have been reported for the direct TNKS–USP25 PPI disruptors C44 or UAT-B. These compounds should therefore be viewed as preclinical chemical-biology probes and proof-of-concept agents rather than clinical candidates. C44 was reported to disrupt the TNKS–USP25 interaction and suppress prostate cancer growth in preclinical models, whereas UAT-B was shown to block TNKS–USP25 complex formation, reduce TNKS abundance, suppress Wnt/β-catenin signaling, and inhibit TNKS-overexpressing colorectal cancer models, including multidrug-resistant settings.^11^

The broader tankyrase-inhibitor field, however, has begun to enter clinical testing. Stenoparib, also known as 2X-121 or E7449, is an orally available dual PARP1/2 and tankyrase 1/2 inhibitor. The National Cancer Institute lists clinical trials using 2X-121, including an active study in recurrent advanced ovarian cancer and a temporarily closed advanced solid tumor study. Stenoparib has also received FDA Fast Track designation for advanced ovarian cancer, and its development incorporates a companion diagnostic strategy to guide patient selection. In addition, basroparib/STP1002 is a tankyrase-selective inhibitor evaluated in a first-in-human phase I dose-escalation study in patients with advanced-stage solid tumors.

These clinical efforts are relevant to the future development of TNKS–USP25 PPI disruptors for two reasons. First, they demonstrate that pharmacological tankyrase targeting can be advanced into human studies, despite earlier concerns regarding Wnt-pathway toxicity and therapeutic window. Second, they provide a useful clinical benchmark for next-generation scaffolding or PPI-directed tankyrase modulators. Unlike catalytic inhibitors, TNKS–USP25 disruptors are designed to reduce TNKS protein stability by blocking USP25-mediated deubiquitination rather than directly inhibiting the PARP catalytic site. Therefore, future clinical translation of TNKS–USP25 disruptors will need to establish not only antitumor activity and safety, but also whether TNKS depletion offers advantages over catalytic inhibition in terms of pathway suppression, biomarker selection, resistance, and tolerability.

## 8. Specificity and safety considerations

### 8.1 What “selectivity” means for tankyrase scaffolding inhibitors

**Fig. 10:**
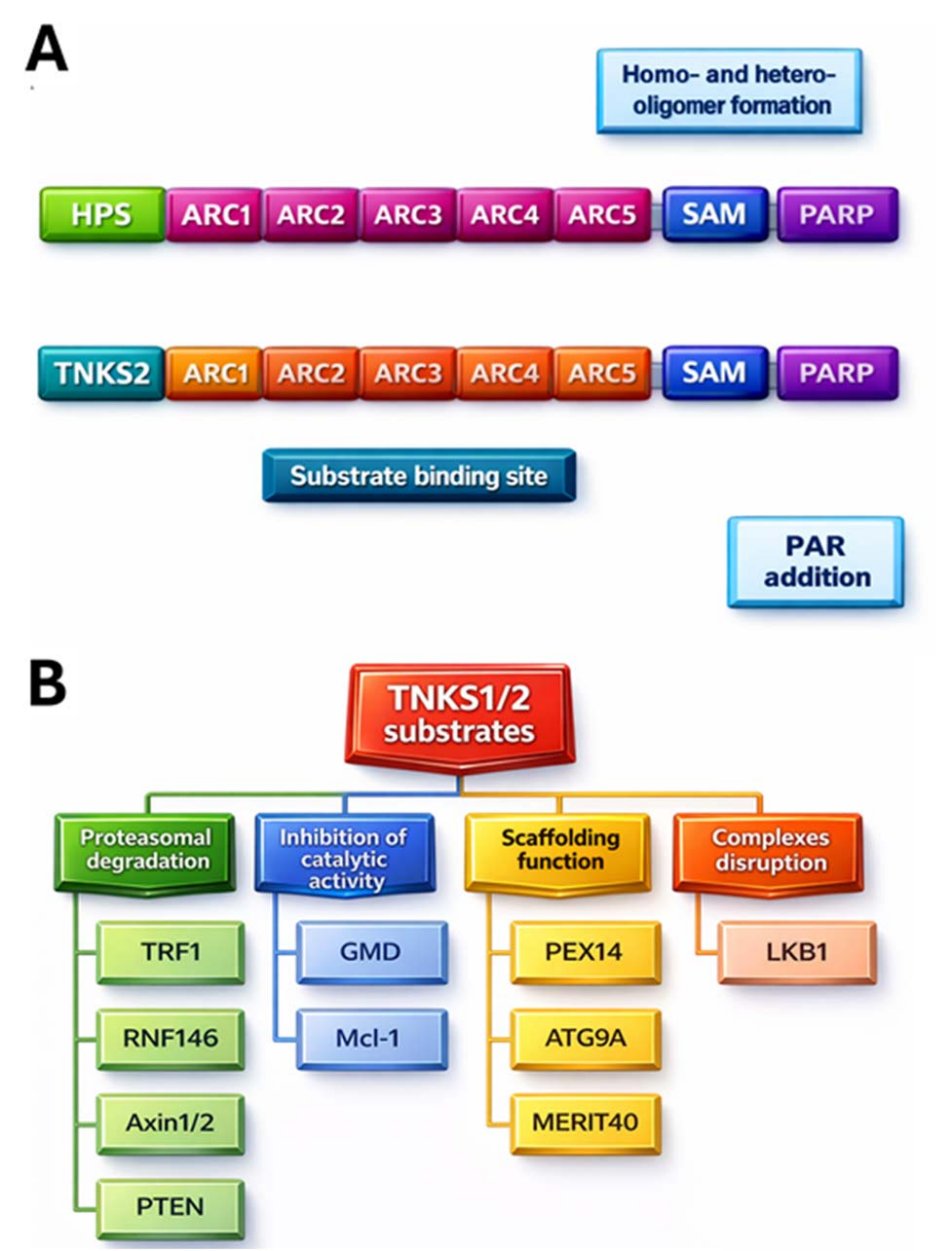
TNKS1/2 structure and functional classification of tankyrase substrates.^35^. (A) Domain architecture of tankyrase 1 (TNKS1) and tankyrase 2 (TNKS2). Both proteins share a similar organization comprising a C-terminal catalytic PARP domain that synthesizes and adds linear poly(ADP-ribose) (PAR) chains onto substrates (PAR addition), a SAM domain that mediates homo- and hetero-oligomer formation, and an N-terminal ankyrin repeat region divided into five ankyrin repeat clusters (ARC1–ARC5) that mediate substrate recognition and binding (with ARC3 generally not involved in substrate binding). TNKS1 additionally contains an N-terminal HPS region of unknown function. (B) Functional categories of representative tankyrase substrates based on the predominant biological outcome associated with their interaction with TNKS1/2: proteasomal degradation (TRF1, RNF146, Axin1/2, PTEN), inhibition of catalytic activity (GMD, Mcl-1), scaffolding function (PEX14, ATG9A, MERIT40), and complexes disruption (LKB1).

For ankyrin-repeat cluster (ARC)-targeting ligands, “selectivity” has at least three layers: (i) paralog selectivity (TNKS1 *vs.* TNKS2), (ii) intraprotein selectivity (which ARC is engaged: ARC1/2/4/5 *vs.* ARC3), and (iii) network selectivity (which subset of TBM-containing substrates are displaced). This differs from catalytic-site inhibitors, where the main selectivity questions are usually TNKS1/2 *vs.* other PARPs and the extent of catalytic blockade.^46^ As illustrated in Fig. 10, tankyrases combine a C-terminal PARP catalytic domain with an extensive N-terminal substrate-recruitment platform built from five ankyrin repeat clusters (ARC1–ARC5), plus a SAM domain that supports multimerization; TNKS1 also contains an additional HPS region not present in TNKS2 (Fig. 9A).^35^ This modular organization helps clarify what “selectivity” means for scaffold-directed (ARC-targeting) ligands: selectivity can occur at the paralog level (TNKS1 *vs.* TNKS2), at the intraprotein level (engaging specific ARCs—typically ARC1/2/4/5 rather than ARC3), and at the network level by preferentially displacing only certain TBM-containing substrates, which themselves can be grouped by downstream outcomes such as degradation, catalytic inhibition, scaffolding, or complex disruption.^35^

Because ARC1/2/4/5 share a conserved TBM-binding groove, many TBM-mimetic ligands are expected to be “pan-ARC” unless deliberate features are introduced to exploit subtle sub-pockets or dynamics.^7^ In contrast, ARC3 is structurally divergent and typically does not recognize canonical TBMs, so activity at ARC3 is neither required nor expected for most substrate-displacement strategies.

### 8.2 ARC5 selectivity *vs.* pan-ARC binding

From a mechanistic perspective, a TNKS–USP25 disruptor does not need to block every ARC; it needs to prevent USP25's TBM-like C-terminus from productively engaging the ankyrin groove(s) that dominate binding in cells. Structural and functional data indicate that USP25 stabilizes tankyrase, increasing TNKS abundance and supporting Wnt/β-catenin output.^10^ Small molecules that block this PPI are therefore often framed as “ARC5 interface” ligands, because ARC5 contains a well-characterized TBM groove that accommodates the USP25 C-terminus.^12^ However, whether a given compound is ARC5-selective (*vs.* ARC1/2/4/5 broadly) is an empirical question. For example, the prostate-cancer tool compound C44 was reported to disrupt the TNKS–USP25 interaction and suppress tumor-associated phenotypes, but the published summary does not establish a comprehensive ARC-by-ARC binding profile.^11^

In contrast, UAT-B provides unusually direct evidence for an ARC5-centered binding mode: in a cellular thermal shift assay, UAT-B stabilized TNKS in cells yet did not inhibit TNKS1/2 enzymatic activity, consistent with a non-catalytic mechanism.^3^ SPR showed direct binding to a TNKS1-ARC5 construct with *K*_D_ ≈ 46.62 µM, and structure-guided mutagenesis pinpointed Asn876 as a key contact—an N876A substitution reduced UAT-B binding and eliminated its ability to block the USP25/TNKS interaction.^10^ These data support a practical workflow for future TNKS–USP25 chemical probes: (1) demonstrate direct biophysics on isolated ARC domains (SPR/ITC/NMR), (2) validate ARC5 (and ideally TNKS1 *vs.* TNKS2) determinants through point mutants, and (3) then quantify how much broader “pan-ARC” displacement occurs in the cellular substrate network.

### 8.3 Implications from ARC4-selective inhibitor work

A major recent advance for specificity is the demonstration that single-ARC selectivity is achievable despite the conserved nature of the TBM groove. Bosetti and colleagues identified a pyrrolone scaffold and optimized S8 (ARCher-142), which binds selectively to TNKS2 ARC4 with ∼8 µM potency and attenuates Wnt/β-catenin signaling in cells.^47^ The structural rationale is especially relevant for ARC5 programs: in the ARC4–S8 co-structure (PDB 9QFC), the compound competes with peptide binding and extends into a hydrophobic sub-pocket, and local loop conformations help rationalize why near-identical ARCs can nevertheless be differentially addressed by small molecules.^47^ Conceptually, ARC4 selectivity suggests ARC5 selectivity may be achievable by identifying (i) ARC5-unique micro-pockets adjacent to the canonical groove, (ii) ARC5-specific loop dynamics that can be stabilized by ligands, and (iii) interaction geometries tuned to the USP25 C-terminus rather than generic TBM consensus.^47^

### 8.4 Off-target risks and how to profile them

For ARC-directed ligands, “off-target” can mean two different things. First, on-target-but-unwanted effects: a pan-ARC ligand may displace many TBM-containing substrates beyond USP25, potentially perturbing telomere maintenance, mitotic regulation, Hippo/YAP regulation, and other tankyrase-linked pathways.^43^ Second, true off-target binding: small molecules can bind unrelated proteins in a way that is independent of tankyrase biology. The ARC4-selective program is instructive because it used counterscreens to filter out assay-interfering compounds during hit identification.^47^ Recommended selectivity profiling for TNKS–USP25 disruptors includes: (i) a panel of ARC1/2/4/5 assays for both TNKS1 and TNKS2 (biophysics and displacement formats), (ii) proteome-wide target ID (thermal proteome profiling or chemoproteomics) to detect unexpected binders, (iii) pathway-anchored readouts that separate USP25-centric effects from broader TBM displacement (*e.g.*, TNKS abundance, AXIN stabilization, Wnt transcriptional reporters), and (iv) standard liability panels (CYPs, hERG) once chemical matter becomes more drug-like.

### 8.5 Safety: what we can infer from catalytic inhibition and what remains unknown

Most preclinical safety knowledge comes from catalytic-site TNKS inhibitors used to suppress Wnt signaling *via* AXIN stabilization. A recurring on-target liability is intestinal toxicity in mice—consistent with the dependence of intestinal crypt homeostasis on Wnt signaling. In a toxicology study of a TNKS inhibitor (G-631), 14 days of dosing caused dose-dependent enteritis and villus damage with a reported therapeutic index <1; some injury was reversible after a recovery period, while more severe exposure led to only partial reversibility and morbidity.^42^ Not all catalytic inhibitors appear equivalent: the TNKS1/2-selective inhibitor STP1002 was reported to show antitumor efficacy in APC-mutant CRC models while exhibiting no significant on-target GI toxicity compared with G007-LK in the tested preclinical settings.^43^ Even in efficacy-focused studies, intestinal toxicity is repeatedly highlighted as a central development challenge for the class.^48^

Additional tissue liabilities have been reported for systemic tankyrase inhibition. For example, tankyrase inhibition has been linked to altered bone homeostasis and bone loss in mouse models (implicating downstream nodes such as SH3BP2).^49,50^ Tankyrase inhibition has also been associated with telomere dysfunction/shortening in cell-based work, consistent with tankyrase roles in telomere regulation.^51,52^ How directly these liabilities translate to TNKS–USP25 PPI disruption is not yet fully established. Mechanistically, PPI disruptors are expected to lower tankyrase abundance (by removing USP25-mediated deubiquitination/stabilization) rather than simply blocking catalysis.^12,53^ Lowering TNKS levels could more fully suppress both catalytic and scaffolding functions, which may broaden efficacy in contexts where TNKS scaffolding contributes to signaling—but it could also intensify on-target toxicity if essential TNKS functions are lost in normal tissues.^12^

### 8.6 Practical recommendations for developing safer, more specific TNKS–USP25 disruptors

Define the desired selectivity early (ARC5-only *vs.* multi-ARC). Use systematic ARC1/2/4/5 displacement panels (TNKS1 and TNKS2) to avoid “hidden” pan-ARC activity.^7,47^ Separate mechanism classes in cells: combine CETSA/TPP for engagement with orthogonal catalytic assays to confirm non-PARP mechanisms (as done for UAT-B).^12^ Prioritize biomarkers that report the intended axis: TNKS abundance and TNKS–USP25 proximity (co-IP/PLA), plus downstream Wnt metrics (AXIN, β-catenin transcriptional reporters).^33,53^ Build safety into the cascade: include intestinal organoids or stem-cell-rich intestinal models, since GI toxicity has repeatedly limited the broader TNKS inhibitor class.^42,43,48^ If systemic exposure is anticipated, profile broader liabilities (bone and telomere) during lead optimization, not after a “winner” emerges.^49–51^

## 9. Therapeutic opportunities

Small-molecule disruption of the tankyrase–USP25 interaction offers a mechanism-based way to lower tankyrase abundance and suppress downstream Wnt/β-catenin outputs. Because the intervention acts upstream of tankyrase catalytic activity—by destabilizing the enzyme *via* blocking USP25-mediated deubiquitination—its potential clinical value is expected to be greatest in settings where tumor growth and/or therapy resistance depend on Wnt/β-catenin signaling and where tankyrase protein is elevated and rate-limiting for pathway output.^11,12^

### 9.1 Patient stratification: Wnt dependency and TNKS/USP25 abundance

Across available PPI-disruptor studies, two practical enrichment variables repeatedly emerge: the degree of Wnt pathway dependence, and the baseline abundance of tankyrase protein. In prostate cancer, aberrant Wnt activation can promote progression and treatment resistance, motivating patient stratification by Wnt pathway activation state rather than tumor site alone.^54^ In MDR CRC models, UAT-B sensitivity tracked with tankyrase expression: higher TNKS levels correlated with lower UAT-B IC50 values (Pearson *r* = −0.7459, *P* = 0.0034; *n* = 13). In the same study, non-malignant cell lines had IC_50_ values >10 µmol L^−1^, whereas a subset of CRC lines had IC_50_ values <10 µmol L^−1^.^12^ These results support a biomarker-driven development path in which TNKS protein level (*e.g.*, IHC score or quantitative proteomics) is used as a candidate predictive biomarker and a pharmacodynamic readout, complemented by functional Wnt outputs (*e.g.*, nuclear β-catenin; Wnt target genes such as AXIN2).^12^

### 9.2 Prostate cancer: proof-of-concept with C44 and Wnt-dependent disease subsets

C44 provides proof-of-concept that an ARC5-focused small molecule can disrupt the TNKS–USP25 interaction and produce an anti-proliferative phenotype in prostate cancer models. C44 was identified by hierarchical virtual screening (>200 000 compounds) against the USP25–TNKS ARC5 complex, and was validated to bind the PPI interface and disrupt complex formation. Mechanistically, C44 increased AXIN half-life and promoted β-catenin breakdown, consistent with suppression of canonical Wnt signaling.^11^ The reported *in vivo* activity of C44 (tumor growth suppression in xenograft models) suggests that PPI disruption can translate beyond biochemical assays, at least in proof-of-concept settings.^11^ Clinically, the most plausible niches are prostate tumors with clear Wnt pathway activation (genetic drivers and/or transcriptional signatures), including advanced disease where Wnt signaling has been linked to progression and therapy resistance. This argues for future preclinical work that pairs TNKS–USP25 disruptors with prostate cancer model stratification by Wnt activation state and TNKS abundance.^54^

### 9.3 Multidrug-resistant colorectal cancer: UAT-B and MDR models

The clearest near-term therapeutic opportunity is MDR CRC. Zhu and colleagues identified UAT-B (a neoantimycin analog) as a TNKS–USP25 PPI inhibitor that decreases TNKS levels and triggers apoptosis through Wnt/β-catenin pathway modulation (Table 2). Transcriptomic and reporter assays supported early Wnt pathway suppression following UAT-B exposure, and CTNNB1 knockdown further linked viability effects to β-catenin dependence.^12^ A clinically relevant feature is activity in drug-resistant settings: UAT-B was evaluated in CRC models resistant to commonly used chemotherapies (including fluoropyrimidine-, platinum-, and anthracycline-based agents) and retained anti-tumor activity in resistant derivatives. This aligns with the authors' broader framing that Wnt/β-catenin signaling is implicated in MDR phenotypes.^12^ A key mechanistic insight is that chemotherapy-resistant CRC lines exhibited increased TNKS and β-catenin relative to parental counterparts, and UAT-B reduced viability in resistant derivatives. In contrast, a representative catalytic tankyrase inhibitor (G007-LK) did not show comparable activity in the resistant setting in this study, highlighting the possibility that lowering TNKS abundance can differ functionally from catalytic blockade.^12^

**Table 2:** Summarizes the reported *in vivo* efficacy of UAT-B across xenograft, PDX, and APCmin/ + models.^12^

Model	Regimen (route)	Efficacy readout	Safety observations reported
SW620 xenograft	UAT-B 0.5 mg kg^−1^ daily × 3 weeks (i.p.)	Tumor inhibition 38.1% (T/C 0.42)	No significant body-weight loss; no major organ pathology
HCT116 xenograft	UAT-B 0.5 mg kg^−1^ daily × 3 weeks (i.p.)	Tumor inhibition 50.4% (T/C 0.30)	No significant body-weight loss; no major organ pathology
PDX (CR3079)	UAT-B 0.5 mg kg^−1^ daily × 3 weeks (i.p.)	Tumor inhibition 59.9% (T/C 0.46)	No significant body-weight loss; no major organ pathology
APCmin/ + intestinal tumors	UAT-B 1 mg kg^−1^ daily × 9 weeks (i.p.)	↓ Tumor burden; ↑ survival	No overt toxicity by weight; no major organ pathology

Collectively, these *in vivo* data prioritize MDR CRC populations enriched for high tankyrase expression and Wnt pathway activity. Given that standard-of-care chemotherapy remains limited by resistance and toxicity, a PPI-disruptor approach that preferentially impacts TNKS-high tumor cells—while sparing non-malignant colorectal cells in vitro—warrants further translational evaluation, including combination testing with chemotherapy backbones.^12^

### 9.4 Broader opportunities and hypothesis-driven extensions

Beyond prostate cancer and CRC, tankyrases have been implicated across multiple tumor contexts *via* both Wnt-dependent and Wnt-independent substrates. Although direct evidence for TNKS–USP25 PPI disruption outside these indications remains limited, the biology supports targeted exploration in tumors where TNKS is overexpressed and contributes to aggressiveness, including lung, breast, gastric, liver, and ovarian cancer contexts summarized in recent reviews.^35^

### 9.5 Practical development considerations: where TNKS levels and Wnt dependence suggest utility

Across indications, the most actionable strategy is to pair PPI disruption with biomarker-defined selection and early pharmacodynamic confirmation. The UAT-B study argues for a therapeutic window because USP25 expression is elevated in tumor tissue relative to adjacent normal tissue, and because UAT-B showed a sensitivity gap between non-malignant and a subset of CRC lines *in vitro*.^12^

## 10. Open questions and future directions

### 10.1 Better ARC5 binders: affinity and permeability

The two reported small-molecule starting points for disrupting the TNKS–USP25 protein–protein interaction (PPI) are (i) C44, a synthetic small molecule linked to anti-proliferative phenotypes in prostate cancer models, and (ii) UAT-B, a natural-product-derived depsipeptide reported to bind the TNKS1 ARC5 domain and to block the TNKS–USP25 interaction in cells.^11,12^ However, the existing chemical matter remains early-stage from a drug-discovery perspective. For example, in surface plasmon resonance (SPR) experiments UAT-B bound a TNKS1 fragment spanning ARC5 (residues 799–981) with a dissociation constant (*K*_D_) of 46.62 µM, suggesting substantial headroom to improve affinity and, likely, cellular target engagement.^12^ The UAT-B study also provides a clear medicinal-chemistry handle: methylation of a phenolic hydroxyl group (UAT-Bp) weakened ARC5 binding (K_D 178.0 µM), consistent with an H-bonding pharmacophore that contributes materially to the interaction energy.^12^

Moving from micromolar binders to a robust chemical probe (and ultimately therapeutic candidates) will likely require parallel optimization of (a) ARC5 affinity (ideally into the low-micromolar or better range in a biophysical assay), (b) permeability and metabolic stability, and (c) selectivity across other TNKS ARCs and across the broader proteome. The latter point is especially important because ARC-binding ligands risk acting as general TBM-site competitors rather than selectively modulating the TNKS–USP25 axis. Practical routes to improved ARC5 binders include fragment-based discovery against ARC5 followed by fragment growing/merging, peptidomimetic approaches that preserve the key TBM-recognition interactions while improving drug-like properties, and macrocycle or constrained-analog strategies to reduce entropic penalties (which can be particularly beneficial for shallow PPI pockets). Precedent that individual ARC domains are ligandable and can be targeted selectively comes from recent ARC4-selective scaffolding inhibitors, supporting the feasibility of an analogous ARC5-focused campaign.^47^

### 10.2 Structure-guided optimization around key ARC5 residues

A key unanswered question is whether ARC5 binders can be optimized in a genuinely structure-guided way without high-resolution structures of ARC5 in complex with either (i) a USP25-derived peptide element, (ii) an ARC5-selective small molecule, or (iii) both. The UAT-B paper used docking plus mutagenesis/biophysics to triangulate an interaction model in which UAT-B forms hydrogen bonds with Arg836, Asn876, and Lys913 on ARC5.^12^ Among these, Asn876 emerges as a particularly informative hotspot: in SPR, the N876A mutation reduced binding dramatically (reported K_D 572.1 µM *vs.* 46.62 µM for wild-type ARC5) and abolished UAT-B's ability to block the USP25/TNKS interaction in cells. Consistent with the same interaction logic, conversion of UAT-B to UAT-Bp (phenolic O-methylation) weakened binding, supporting the idea that a hydrogen bond to the N876 region is a critical pharmacophore.^12^

These observations suggest several concrete next steps for structural optimization and mechanism validation: Obtain ARC5–ligand co-structures (X-ray crystallography or cryo-EM/NMR-guided models) to define tractable vectors for affinity gains. Docking-derived hypotheses can be rapidly refined once an experimental structure reveals water networks, side-chain conformational freedom, and adjacent hydrophobic subpockets. Use an N876-dependence filter as an on-target triage: analogs should lose binding and cellular PPI-disruption activity against N876A (or related) ARC5 mutants, providing a stringent control for ARC5 engagement. Systematically explore H-bond donors/acceptors and steric bulk around the phenolic pharmacophore to maximize interactions with the N876 region while reducing polar surface area (to improve permeability). Map ARC5 *vs.* other ARC binding pockets experimentally (*e.g.*, using panel SPR/DSF/NMR assays for ARC1–ARC5) to avoid drifting into pan-ARC TBM-site competitors during optimization.

### 10.3 Degraders and dual-mechanism agents: combining catalytic and scaffolding targeting

A recurring theme in the tankyrase field is that catalytic inhibition does not always phenocopy tankyrase depletion. Tankyrase catalytic inhibitors can stabilize AXIN, but they can also increase TNKS abundance; this may amplify TNKS scaffolding functions and, in some contexts, blunt suppression of beta-catenin turnover. A 2025 preprint reported that catalytic inhibition promoted accumulation of TNKS within the destruction complex and drove formation of AXIN puncta that impaired beta-catenin turnover, whereas chemically induced TNKS degradation (using the degrader IWR1-POMA, reported DC50 ∼60 nM) avoided puncta formation and produced deeper suppression of WNT/beta-catenin signaling and colorectal cancer cell proliferation.^41^

This has two important implications for TNKS–USP25 PPI disruption:

• If disrupting TNKS–USP25 lowers TNKS levels (by preventing USP25-mediated deubiquitination/stabilization), PPI disruption could in principle shift the biology toward the ‘degradation-like’ state rather than the ‘catalytic-inhibition-like’ state. UAT-B is consistent with this model: it blocked TNKS–USP25 association and was associated with reduced TNKS protein levels in cells.

• Dual-mechanism strategies become attractive. One option is combination therapy (an ARC5 PPI disruptor plus a catalytic inhibitor) to decouple AXIN stabilization from undesirable TNKS accumulation. Another, more ambitious option is a single molecule that links a catalytic-domain binder to an ARC5 binder (bivalency) or that uses either warhead to recruit an E3 ligase and degrade TNKS (PROTAC-style).

Evidence supporting these ideas can be found in UAT-B mechanistic experiments (co-IP/PLA disruption of TNKS–USP25; SPR binding to ARC5; and reduced TNKS levels upon treatment in USP25-overexpressing CRC cells).^12^ Finally, any move toward *in vivo* translation will need to navigate the tolerability concerns that have historically limited tankyrase catalytic inhibitors, particularly gastrointestinal toxicity in Wnt-dependent normal tissues. Recent catalytic inhibitors such as STP1002 and OM-153 have been reported to show preclinical antitumor activity with improved GI tolerability/therapeutic windows, offering useful benchmarks for what ‘safe enough’ might look like and providing reference pharmacology for combination or comparison studies.^43,55^ In parallel, broader mechanistic work is still needed to predict context-dependent outcomes of tankyrase manipulation (catalytic inhibition, scaffolding inhibition, or depletion) across different pathways and tissues; recent reviews highlight that this knowledge gap is itself a major obstacle to clinical translation.^56^

### 10.4 Biomarkers and patient selection for TNKS–USP25 axis targeting

A practical translational question is how to identify settings where TNKS–USP25 PPI disruption is most likely to be effective. In TNKS-overexpressing colorectal cancer models, UAT-B sensitivity correlated inversely with baseline TNKS protein levels across cell lines (reported Pearson *r* = −0.7459), suggesting that TNKS abundance itself could serve as a pharmacodynamic/selection biomarker for ARC5-targeting strategies.^12^ More broadly, future studies should test whether TNKS levels, USP25 expression/activity, and readouts of WNT pathway dependence (*e.g.*, AXIN turnover, beta-catenin target gene expression) jointly predict response, and whether these relationships hold across other Wnt-driven contexts (including prostate cancer models implicated in the C44 study).^11^

## 11. Conclusion

Disrupting the tankyrase–USP25 protein–protein interaction is emerging as a distinct and potentially advantageous way to modulate tankyrase-dependent signaling. Unlike catalytic tankyrase inhibition, which primarily suppresses poly(ADP-ribose) synthesis while leaving the multi-domain scaffold intact, TNKS–USP25 PPI disruption aims upstream at tankyrase stability. By preventing USP25-mediated deubiquitination, this strategy can reduce cellular tankyrase abundance, thereby reshaping both catalytic and non-catalytic (scaffolding) outputs that support Wnt/β-catenin signaling and other tankyrase-regulated pathways. The field now has a coherent mechanistic chain linking structure to function: the USP25 C-terminus contains a short tankyrase-binding element that engages the ankyrin repeat clusters (ARCs), and ARC5 provides a well-defined binding pocket with recurring hotspot features that can be targeted by ligands. The availability of high-resolution structural information for ARC–peptide recognition, together with assay toolkits that quantify binding and displacement, has made this interaction a tractable target for rational discovery and optimization.

Proof-of-concept studies have also begun to connect TNKS–USP25 disruption with therapeutically meaningful phenotypes. Reported small-molecule disruptors demonstrate that interfering with ARC5-centered binding can decrease tankyrase levels, stabilize negative regulators of Wnt signaling, and suppress tumor growth in relevant models. These results support the broader premise that “de-stabilizing the scaffold” can achieve pathway control that is mechanistically complementary to blocking the PARP catalytic site. At the same time, TNKS–USP25 PPI disruption raises challenges that will shape the next phase of research. First, achieving robust potency in cells will require improvements in affinity and permeability without sacrificing selectivity across ARC domains and across unrelated protein–protein interfaces. Second, the biological consequences of lowering tankyrase levels are context-dependent, because tankyrases integrate multiple interaction networks; careful biomarker strategies will therefore be essential to identify settings where tankyrase abundance and Wnt dependence predict response. Third, safety will hinge on minimizing off-target activity and avoiding excessive suppression of tankyrase functions in tissues where homeostatic signaling is required.

Looking forward, the most promising opportunities lie at the intersection of structure-guided chemistry and modality innovation. Systematic exploitation of ARC5 pocket determinants, guided by co-structures and mutational sensitivity, should enable more selective and durable binders. Parallel development of dual-mechanism approaches—such as combining PPI disruption with catalytic inhibition, or leveraging the ARC interaction to drive targeted protein degradation—could further widen the therapeutic window by tuning the depth and duration of tankyrase pathway suppression. As these chemical and biological tools mature, TNKS–USP25 PPI disruption is well positioned to move from a compelling concept to a broadly useful strategy for interrogating tankyrase biology and, potentially, for treating Wnt-driven and tankyrase-dependent disease.

## Author contributions

E. M. K. and F. F. A.: conceptualization, writing – original draft, writing – review & editing, supervision, methodology, and investigation. S. M. K. and M. A.: project administration, funding acquisition, methodology, and investigation formal analysis, validation, supervision, methodology, and investigation, N. A. A.: supervision, methodology, investigation, software, and visualization. A. M. L. and S. A.: resources, writing – review & editing, supervision formal analysis, validation, supervision, methodology, and investigation.

## Conflicts of interest

Authors declare no conflicts of interest.

## Data Availability

No primary research results, software or code have been included and no new data were generated or analyzed as part of this review
